# SARS-CoV-2 infection and vaccination elicit distinct pharyngeal mucosal B cell responses in children

**DOI:** 10.21203/rs.3.rs-7428491/v1

**Published:** 2025-09-09

**Authors:** Kalpana Manthiram, Qin Xu, Lihong Shi, Liya Wang, Foo Cheung, Aparna Kotekar, Galina Koroleva, Elizabeth Rice, Richard Apps, Justin Lack, Craig Martens, Iyadh Douagi, Can Liu, Juraj Kabat, Hengameh Behzadpour, Lela Kardava, Tovah Markowitz, Margery Smelkinson, Kenneth Hoehn, Clarisa Buckner, Dominic Golec, Lorenza Bellusci, Gabrielle Grubbs, Sara Pourhashemi, Juanjie Tang, Asya Khleborodova, Martha Kirby, Rachel Sparks, Andrew Martins, John Tsang, Susan Moir, Surender Khurana, Pamela Mudd, Pamela Schwartzberg

**Affiliations:** NIAID/National Institutes of Health; NIAID/National Institutes of Health; National Institute of Allergy and Infectious Diseases; National Institute of Allergy and Infectious Diseases; Trans-NIH Center for Human Immunology, Autoimmunity, and Inflammation (CHI); National Institute of Allergy and Infectious Diseases; NIAID/National Institutes of Health; National Institute of Allergy and Infectious Diseases; University of Cambridge; National Institute of Allergy and Infectious Diseases; NIH; NIH; Yale School of Medicine; National Institutes of Health; Children’s National Hospital; National Institute of Allergy & Infectious Disease; NIAID, NIH; NIAID; Department of Biomedical Data Science, Geisel School of Medicine at Darmouth, Hanover, NH, United States; National Institutes of Health; National Institute of Allergy and Infectious Diseases; Food and Drug Administration; Food and Drug Administration; Food and Drug Administration; CBER/Food and Drug Administration; National Institute of Allergy and Infectious Diseases; National Human Genome Research Institute; National Institute of Allergy and Infectious Diseases; Yale University; Yale University; Laboratory of Immunoregulation, National Institute of Allergy and Infectious Diseases, National Institutes of Health; Food and Drug Administration; Children’s National Hospital; NIAID/National Institutes of Health

## Abstract

Mucosal immunity is an important correlate of protection against respiratory infections such as SARS-CoV-2. Comparing B cell responses in the upper respiratory tract following vaccination and infection may offer unique insights into mucosal immunity. Here, we characterized antigen-specific B cells in the tonsils, adenoids, and peripheral blood of children who had been infected with SARS-CoV-2 or vaccinated with SARS-CoV-2 mRNA vaccines. SARS-CoV-2-specific switched memory B cells (B_SM_) and germinal center B cells were found in the blood and pharyngeal lymphoid tissues after vaccination or infection. However, infection generated a higher proportion of IgA^+^ B_SM_ and CXCR3^+^CD21^+^ B_SM_, which showed distinct spatial localization, greater clonal expansion and increased propensity for plasma cell differentiation compared to their CXCR3^−^ counterparts, accompanied by persistent activation of innate and T follicular helper cells in the tissues. Our data provide evidence for tissue-specific B cell memory after either SARS-CoV-2 vaccination or infection, but with distinct characteristics that can influence the quality, durability, and localization of immunity.

## INTRODUCTION

The high mortality and far-reaching effects of the COVID-19 pandemic triggered the rapid development of several vaccine platforms including two mRNA-based vaccines, BNT162b2 (Pfizer) and mRNA-1273 (Moderna), which were shown to have high efficacy in preventing severe COVID-19^1, 2^. These vaccines generate high-titers of serum antibodies with neutralizing activity against early and more later circulating SARS-CoV-2 strains, as well as virus-specific memory B and T cells in the peripheral blood^3, 4, 5^. However, the immunity afforded by these vaccines wanes with time and population-based studies suggest that the durability of immune protection following vaccination may be lower than that from prior infection with SARS-CoV-2^6, 7, 8, 9^. SARS-CoV-2 initially enters and infects upper respiratory tract tissues^10, 11^, and immunity in the upper respiratory tract, particularly mucosal IgA levels, has been shown to correlate with protection against COVID-19^12, 13^. Therefore, characterizing upper respiratory tract immunity provided by these intramuscularly delivered vaccines is important to understand their efficacy and to provide insights for future vaccine development against a variety of pathogens.

A major component of protective immune memory to viruses, including SARS-CoV-2, is the development and maintenance of long-lasting, high-affinity memory B cells, which are poised to rapidly differentiate into antibody-secreting cells upon secondary antigen encounter^14, 15^. A subset of memory B cells in the tissues, known as tissue-resident memory B cells, do not recirculate and have been shown to provide effective localized immunity^16^. Secondary lymphoid organs enable the development of germinal centers (GC), which are essential for creating long-lived and high-affinity memory B cells. In these specialized structures, B cells undergo somatic hypermutation (SHM) and affinity maturation with help from T follicular helper (Tfh) cells. COVID-19 mRNA vaccines have been shown to generate SARS-CoV-2-specific Tfh and GC B cells (GCBs) in the draining axillary lymph nodes, which can persist at least 6 months^17, 18, 19, 20^; however, the ability of intramuscular vaccines to elicit immunity in upper respiratory tract lymphoid tissues remains an important question. Furthermore, whether immune memory in the tissue differs after vaccination versus infection is largely unknown.

The tonsils and adenoids are secondary lymphoid structures at the mucosal surface of the upper respiratory tract that contain lymphoid cells not found in the peripheral blood; viral-specific GCBs and tissue-resident memory T and B cells have been described in these mucosal tissues following respiratory infections^21, 22, 23^. Assessing SARS-CoV-2-specific-B cells in these tissues following intramuscular vaccination may, therefore, offer insights into the characteristics, magnitude, and durability of tissue immunity at the sites where the host first encounters the virus.

Here, we characterized SARS-CoV-2-specific B cells in the tonsils, adenoids, and peripheral blood of children immunized with a SARS-CoV-2 mRNA vaccine compared with those of children previously infected with SARS-CoV-2, taking advantage of the unique period during the COVID-19 pandemic to study immunity to an unencountered airborne pathogen and new vaccines. We found SARS-CoV-2-specific B cells, which were primarily switched memory B cells (B_SM_) but also some GCBs, in the tonsils and adenoids of children both post-infection and post-vaccination, indicating that immune memory can be found and maintained in the upper respiratory tract after intramuscular vaccination as well as after infection. Nonetheless, vaccination and infection generated B_SM_ with different characteristics, including increased induction of a population defined by CXCR3 and CD21 expression post-infection. This CXCR3^+^CD21^+^ B_SM_ population showed a greater propensity for plasma cell differentiation and mucosal homing, had unique spatial distribution, and correlated with persistent adaptive and innate immune cell activation in mucosal lymphoid tissues. Our findings provide a framework for understanding responses to vaccination and infection, including novel parameters for assessing vaccine-induced mucosal immunity.

## RESULTS

### SARS-CoV-2-specific B cells are found in the pharyngeal tissues and blood post-vaccination

**Figure 1:**
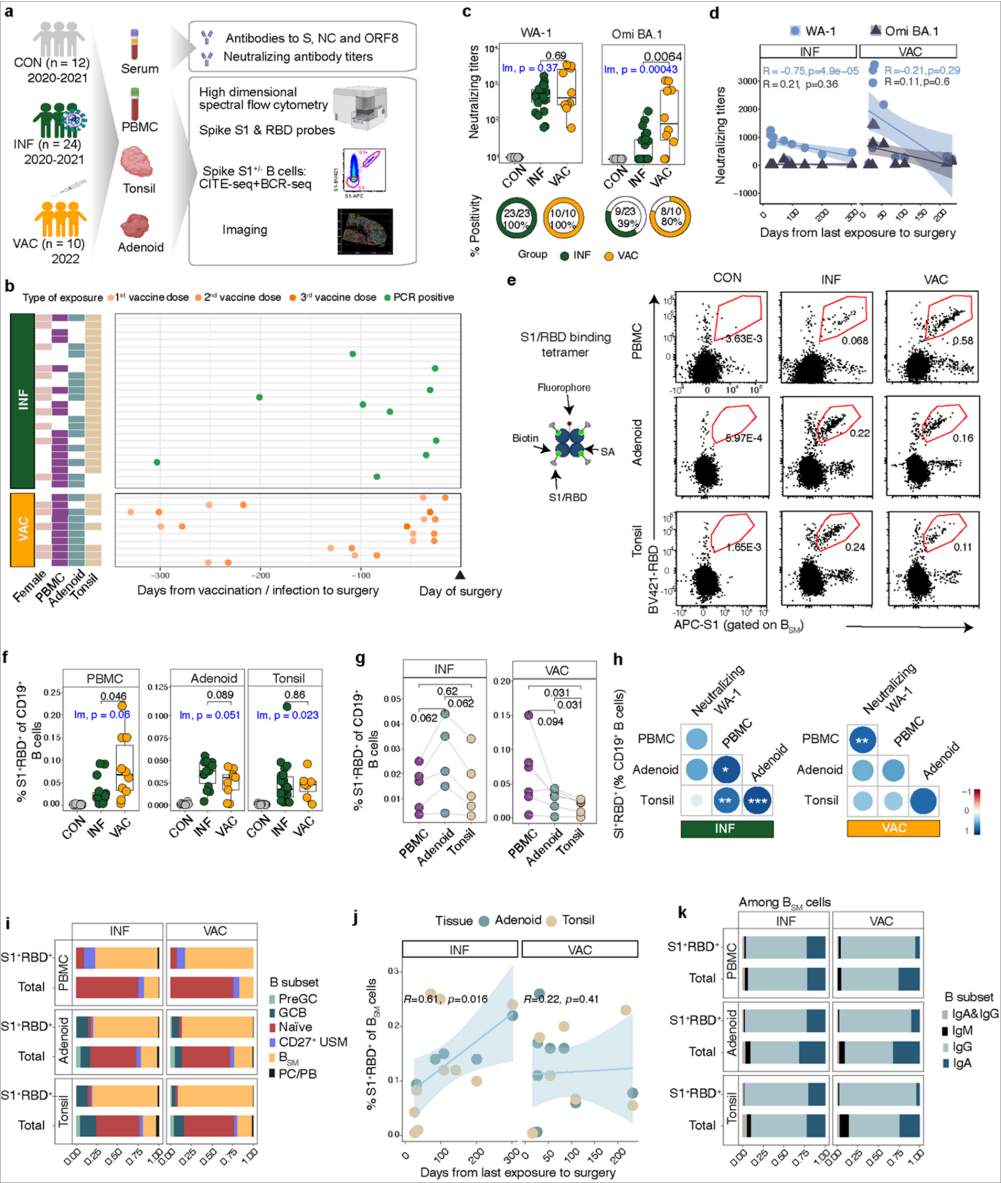
SARS-CoV-2-specific B cells are found in the pharyngeal tissues and blood post-vaccination (a) Study design and participant enrollment. CON (control), INF (post-infection), VAC (vaccinated). S = spike, NC = nucleocapsid, ORF8 = open reading frame 8 (b) Time from vaccination(s) (shown in orange) and/or infection (shown in green) to surgery (right). Samples received for each participant and sex of participant (left). (c) Upper panel: serum neutralizing antibody titers (PsVNA50) against the early strain WA-1 and later circulating strain B1.1.159 (omicron, BA.1) (CON N = 12, INF N = 23, VAC N = 10). Lower panel: Proportion of subjects with neutralizing antibodies against the indicated strain in INF and VAC groups. (d) Correlation between serum neutralizing antibody titers to WA-1 or omicron BA.1 and days from last exposure (infection or vaccination) to surgery (INF N = 10, VAC N = 9) with Spearman’s test. 95% confidence intervals are shaded. (e) Flow cytometry plots showing the percentage of S1^+^RBD^+^ cells (stained with fluorescently labelled S1 and RBD tetramers) among B_SM_ in PBMCs, adenoids, and tonsils of representative CON, INF and VAC participants. (f) Percentage of S1^+^RBD^+^ cells among CD19^+^ B cells in PBMCs, adenoids, and tonsils of CON, INF and VAC participants (PBMC: CON N = 12, INF N = 15, VAC N = 10; adenoid: CON N = 10, INF N = 14, VAC N = 9; tonsil: CON N = 12, INF N = 22, VAC N = 7). (g) Percentage of S1^+^RBD^+^ cells among CD19^+^ B cells in matched PBMCs, adenoids, and tonsils from INF (N = 5) versus VAC (N= 6) participants, compared with two-sided Wilcoxon signed ranks test (paired). (h) Correlations among serum neutralizing titers to WA-1 and percentage of S1^+^RBD^+^ B cells in PBMCs, adenoids, and tonsils. INF (left) and VAC (right) are shown separately (PBMC INF N = 9, VAC N = 10; tonsil INF N = 10, VAC N = 7; adenoid INF = 5, VAC = 9). Correlations assessed with Spearman’s rank correlation. Correlation coefficients (r values) are indicated by colors shown in the bar. Size of the circle indicates absolute r value. Significant p values are indicated as * p<0.05, ** p<0.01, *** p<0.001. (i) Composition of S1^+^RBD^+^ B cells and total CD19^+^ B cells in PBMCs, adenoids, and tonsils of INF and VAC determined by ow cytometry. Samples with fewer than 25 S1^+^RBD^+^ B cells were excluded from this figure. Mean frequencies are shown (PBMC: INF N = 11, VAC N = 9; adenoid: INF N = 14, VAC N = 8; tonsil: INF N = 22, VAC N = 6). B_SM_ = switched memory B cells, USM = unswitched memory B cells, GCB = germinal center B cells, PC/PB = plasma cells/plasmablasts. (j) Correlation between percentage of S1^+^RBD^+^ cells among B_SM_ in adenoid and tonsil and days from last exposure to surgery in INF and VAC groups (adenoid: INF N = 5, VAC N = 9; tonsil: INF N = 10, VAC N = 7) with Spearman’s test. 95% confidence intervals are shaded. (k) Immunoglobulin isotype composition among S1^+^RBD^+^ B_SM_ cells and total B_SM_ in PBMCs, adenoids, and tonsils of INF and VAC. Samples with < 10 S1^+^RBD^+^ B_SM_ were excluded (PBMC: INF N = 13, VAC N = 9; adenoid: INF N = 14, VAC N = 8; tonsil: INF N = 22, VAC N = 6). Mean frequencies are shown (Supplemental Table 4). P values obtained from linear model in panels c and f correcting for participant age (in blue) or from two-sided Mann-Whitney U test (in black) are shown. Medians ± quartiles are shown in box plots. *P* < 0.05 were considered significant.

To assess SARS-CoV-2-specific immunity in vaccinated individuals, we collected serum, peripheral blood mononuclear cells (PBMC), and tonsil and adenoid tissues from 21 children undergoing tonsillectomy and/or adenoidectomy from December 2021 to September 2022 at Children’s National Hospital in Washington, DC, USA, who had received at least one dose of the monovalent mRNA vaccines, BNT162b2 or mRNA-1273 (Figure 1a, Supplemental Tables 1 and 2). These vaccines encode the ancestral Wuhan strain-like spike protein. Of these vaccinated subjects, 10 had no evidence of prior SARS-CoV-2 infection by history of positive PCR or antigen test, nor by positive serum titers against nucleocapsid (NC) and/or open reading frame 8 (ORF8), which have been used for serodiagnosis of SARS-CoV-2 infection^24, 25^ (Supplemental Table 3). These 10 subjects comprised our vaccinated only cohort (VAC).

We compared these vaccinated children to subjects we previously recruited from late 2020 to early 2021 who had prior SARS-CoV-2 infection (INF), before the availability of vaccination for children (Figure 1a, Supplemental Tables 1 and 2)^23^. All had mild or asymptomatic infection. Based on the timing of sample collection and known infection dates, most of these subjects were likely infected with the D614G or alpha strains, which bear sequence similarity to the spike mRNA used in the BNT162b2 or mRNA-1273 vaccines administered to the VAC cohort. The interval from infection to surgery among INF participants was comparable to the interval from the last vaccination to surgery in the VAC group (Figure 1b, Extended Data Figure 1a). We also included a group of unvaccinated pediatric controls (CON) with no serologic or cellular evidence of prior COVID-19, who were recruited during our initial study (Figure 1a, Supplemental Table 2 and 3). To compare VAC and INF groups, we used both direct statistical comparisons (Mann-Whitney U) and linear models correcting for age.

All VAC and INF participants had serum neutralizing antibodies to the WA-1 strain (Figure 1c, Supplemental Table 3) with no significant differences noted between the two groups. Similar to our INF cohort, VAC subjects showed a trend towards lower neutralizing titers to WA-1 with greater time from infection (Figure 1d), consistent with previous reports^26^. We also evaluated neutralizing titers to omicron, a variant that emerged in late 2021 and rapidly became the dominant strain, causing numerous breakthrough infections in those who were previously infected or vaccinated^27, 28^. Although neutralizing titers were lower to omicron than to WA-1 in both groups, they were on average higher in VAC than INF, with a higher proportion of VAC subjects having positive titers (Figure 1c, Supplemental Table 3).

We then used fluorescently-labeled probes for the receptor-binding domain (RBD) and S1 portion of the spike protein from the original Wuhan strain to identify SARS-CoV-2-recognizing B cells (S1^+^RBD^+^) by ow cytometry (Figure 1e). As previously reported, S1^+^RBD^+^ CD19^+^ B cells were found in both tissues and blood of most INF participants (Supplemental Table 3). Notably, almost all VAC subjects also had S1^+^RBD^+^ CD19^+^ B cells in both their pharyngeal tissues and blood (Figure 1f, Supplemental Table 3). A higher frequency of S1^+^RBD^+^ cells was noted among B cells in peripheral blood of VAC compared to INF individuals but these trended towards a higher frequency in the adenoids of INF subjects (Figure 1f). These findings were confirmed comparing S1^+^RBD^+^ B cells in the blood versus tissues within individual subjects by group (Figure 1g). Among INF subjects, the percentage of S1^+^RBD^+^ B cells in the PBMCs, adenoids, and tonsils were all significantly correlated, as previously reported (Figure 1h)^23^. In contrast, among VAC subjects, the percentage of S1^+^RBD^+^ B cells in the PBMCs correlated significantly with serum neutralizing titers, but not significantly with the percentages of S1^+^RBD^+^ B cells in the adenoid and tonsil (Figure 1h).

We also identified omicron-recognizing B cells (RBD-Omi^+^) using two fluorescently labelled RBD probes from the omicron strain (Extended Data Figure 1b-c). VAC participants had a higher proportion of detectable S1^+^RBD^+^ B cells that also recognized omicron in the blood, adenoid and tonsil compared to INF (Extended Data Figure 1d), suggesting vaccination provides broader coverage of variants than infection in both the blood and tissues^4, 19, 29^. Thus, although vaccinated children had greater B cell responses in the peripheral blood compared to the mucosal tissues, we could detect SARS-CoV-2-specific B cells in the secondary lymphoid tissue of the upper respiratory tract, distal from the site of intramuscular immunization in nearly all vaccinated children.

### Infection induces a higher proportion of IgA^+^ SARS-CoV-2-specific B_SM_ than vaccination

To provide insight into SARS-CoV-2-specific B cells, we used a high dimensional ow cytometry panel consisting of 29 markers including fluorescently labelled SARS-CoV-2 probes as well as surface markers to describe B cell subsets, isotype, activation, tissue residence, and homing. In both INF and VAC, the majority of S1^+^RBD^+^ B cells in PBMCs, tonsils, and adenoids were B_SM_ (Figure 1i). Unlike neutralizing titers which declined with greater time from vaccination/infection, the percentage of S1^+^RBD^+^ B_SM_ in the tissues were stable (VAC) or increased (INF) with time (Figure 1j).

Mucosal IgA is protective against SARS-CoV-2 infection, but mRNA vaccination has been shown to engender less mucosal IgA in the respiratory tract compared to infection^9, 12, 30, 31^. SARS-CoV-2-specific B_SM_ were predominantly IgG^+^ post-infection and post-vaccination (Figure 1k). However, INF individuals had a greater proportion of IgA^+^ cells (and less IgG^+^ cells) among S1^+^RBD^+^ B_SM_ in PBMC and tissues compared to VAC subjects (Figure 1k, Extended Data Figure 1e). Thus, infection with early circulating strains was associated with a greater proportion of IgA^+^ B_SM_ than vaccination, both in tissues and blood.

In humans, B_SM_ can be subdivided based on expression of CD21 and CD27^32, 33^. CD27^+^CD21^+^ conventional or “resting” memory B cells (cMBCs) are quiescent, highly affinity-matured cells that emerge from GC reactions. Following infection or vaccination, responding B cells become activated and display heterogenous phenotypes including downregulation of CD21^15, 34, 35, 36 37, 38^. Those that express CD27 (CD27^+^CD21^−^) are referred to as activated memory cells (acMBC), while those that express low CD21 and CD27 are called atypical MBCs (atMBC); both are expanded in chronic infections, autoimmune diseases, and shortly after acute infection and vaccination including COVID-19 vaccines^35, 38, 39, 40, 41, 42, 43, 44^. The atMBCs (and some acMBCs) express CD11c, FCRL3, FCRL4, FCRL5, and CD85J, as well as Tbet^45, 46, 47^.

Most of the SARS-CoV-2-specific B_SM_ in both VAC and INF tissues and blood were cMBC (Extended Data Figure 1f-g, Supplemental Table 4). However, a larger proportion of acMBCs were noted in VAC PBMC and tonsils compared to INF, with a similar trend in the adenoids (Extended Data Figure 1f-g). Neutralizing titers correlated with the proportion of acMBCs among S1^+^RBD^+^ B_SM_ in both blood and adenoids (Extended Data Figure 1h). Furthermore, like neutralizing titers, the frequency of CD21^lo/−^ B_SM_ populations (acMBC and atMBC) in the blood declined with time from vaccination (Extended Data Figure 1i).

### SARS-CoV-2-specific B_SM_ in the blood are phenotypically different post-infection and post-vaccination

To further compare the phenotypes of S1^+^RBD^+^ B cells between the two groups beyond traditional B_SM_ markers, we concatenated the S1^+^RBD^+^ B cells from all VAC and INF participants and performed unsupervised clustering analyses based on expression of 19 B cell surface markers. Cells from the peripheral blood and tissues were assessed separately due to differences in cell populations.

**Figure 2:**
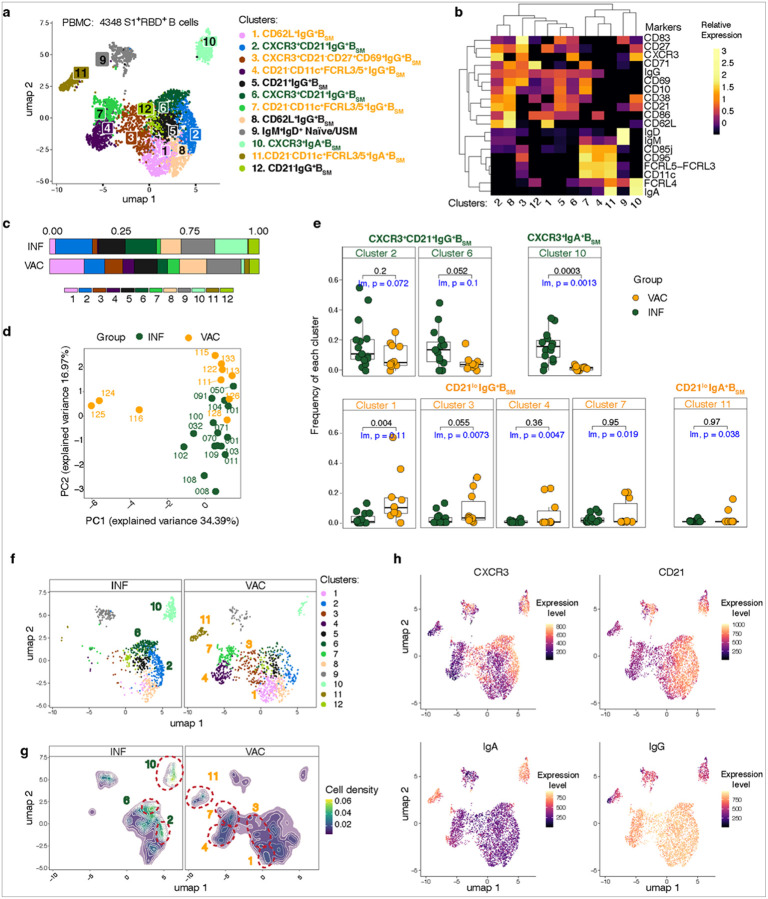
Unsupervised analyses of S1^+^RBD^+^ B cells in the peripheral blood (a) Uniform manifold approximation and projection (UMAP) of unsupervised clustering based on surface markers from flow cytometric analysis of 4348 S1^+^RBD^+^ CD19^+^ B cells of PBMC from both INF (1275 cells) and VAC (3073 cells) groups. Clusters enriched in VAC group are indicated in yellow and INF group in green in the legend. (b) Heatmap of marker/antibody expression in each cluster. (c) Mean proportion of each cluster across subjects among S1^+^RBD^+^ B cells in each group. (d) Principal component analysis (PCA) of each subject based on cluster frequencies. Each point represents an individual and is colored by group. (e) Comparison of frequencies of each cluster among S1^+^RBD^+^ B cells by group. Differentially distributed clusters are shown; data from all clusters are shown in Extended Data Figure 2a. Medians ± quartiles are shown in box plots. P values obtained from linear model correcting for participant ages (in blue) and from two-sided Mann-Whitney U test (in black) are shown. *P* < 0.05 was considered significant. (f) Distribution of S1^+^RBD^+^ B cells among clusters by group on UMAP. (g) Densities of S1^+^RBD^+^ B cells in each cluster by group, with density shading indicating the concentration of cells in different areas of UMAP. Color represents cell density. (h) Heatmaps of selected individual marker/antibody expression overlayed on UMAP. For all panels, INF N = 15, VAC N = 10 (Supplemental Table 5).

Unsupervised clustering of S1^+^RBD^+^ B cells from PBMCs generated 12 phenotypically distinct clusters, 11 of which were B_SM_ subsets (Figure 2a–c, Supplemental Table 5). VAC and INF samples largely segregated on principal component analysis (PCA) on PC2; a few VAC subjects were distinct on PC1 (Figure 2d). We compared the proportion of each cluster in the two groups by Mann-Whitney U or a linear model correcting for age (Figure 2c, e; Extended Data Figure 2a), and found that INF subjects had higher proportions of S1^+^RBD^+^ B cells in cluster 10 with a trend towards more in clusters 2 and 6 compared to VAC individuals; these three clusters represented CXCR3^+^ B_SM_ (Figure 2b, e–h). Cluster 10, the most significant cluster, represented IgA^+^CXCR3^+^ B_SM_. CXCR3 is a chemokine receptor induced by IFN-g that directs B_SM_ to areas of in ammation^48^. Although CXCR3 can be expressed on CD21^−^ atMBCs along with CD11c and other markers regulated by Tbet^49, 50^, these three clusters did not express atMBC markers and were largely CD21^+^ (Figure 2a–b, h)^5, 42^.

Conversely, VAC subjects had a higher portion of S1^+^RBD^+^ B cells in several CD21^lo^ clusters, including clusters 1, 3, 4 and 7 (CD21^lo^IgG^+^ B_SM_), and cluster 11 (CD21^lo^IgA^+^ B_SM_ cluster found in 2 subjects) (Figure 2a–c, e–h). Clusters 4, 7 and 11 expressed atMBC markers including CD11c, CD85J, FCRL3/5 and CD95 and had a mixture of CD27^+^ and CD27^−^ cells but were not CXCR3^+^ (Figure 2b). Cluster 3 was an IgG^+^ CD21^−^ CD27^+^ acMBC cluster that expressed variable CXCR3. Thus, unbiased analyses revealed phenotypic differences in SARS-CoV-2-specific B cells in the peripheral blood post-infection and post-vaccination, including enrichment of CXCR3^+^CD21^+^ B_SM_ post-infection.

### Distinct SARS-CoV-2-specifc GCBs and B_SM_ are in the pharyngeal tissues of INF and VAC subjects

**Figure 3:**
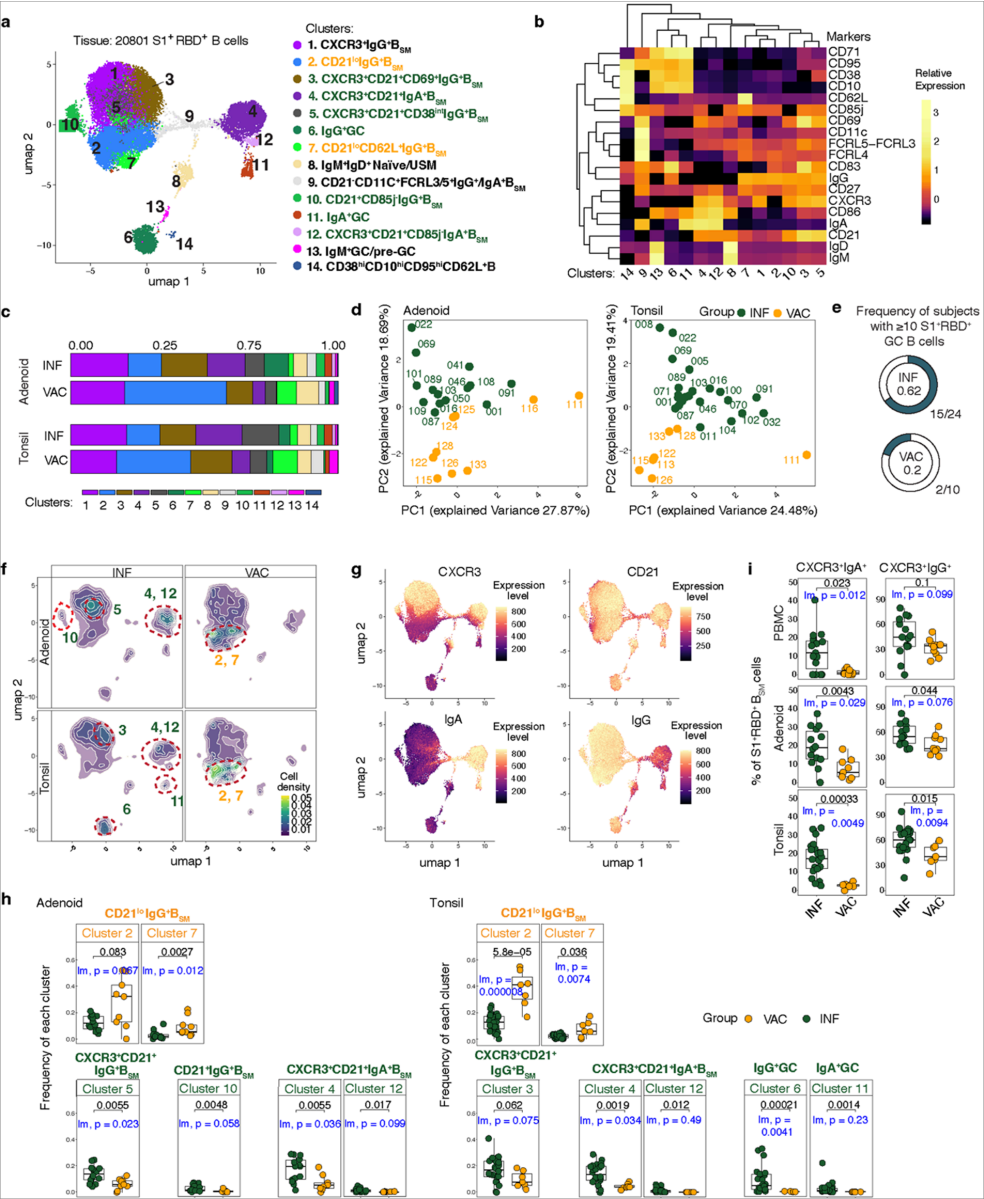
Unsupervised analyses of S1^+^RBD^+^ B cells in the adenoid and tonsil (a) UMAP of unsupervised clustering based on surface markers from ow cytometric analysis of 20,801 S1^+^RBD^+^ CD19^+^ B cells from adenoid and tonsil of both INF (14,627 cells) and VAC (6174 cells) subjects. Clusters enriched in VAC group are indicated in yellow and INF group in green in legend. (b) Heatmap of marker/antibody expression in each cluster. (c) Mean proportion of each cluster across subjects among S1^+^RBD^+^ B cells in INF and VAC tissues. (d) PCA of each subject based on cluster frequency. Each point represents an individual and is colored by group. (e) Frequency of subjects with ≥ 10 S1^+^RBD^+^ GCB cells in at least one tissue in INF and VAC participants (see Supplemental Table 7 for details). (f) Densities of S1^+^RBD^+^ B cells in each cluster by group, with density shading indicating the concentration of cells in different parts of the UMAP. Color represents cell densities. (g) Heatmaps of selected individual marker/antibody expression overlayed on UMAP. (h) Comparisons of cluster frequencies among S1^+^RBD^+^ B cells in INF and VAC groups according to tissue type. Differentially distributed clusters are shown; data from all clusters are shown in Extended Data Figure 2d. Medians ± quartiles are shown in the box plots. (i) Percentage of CXCR3^+^IgA^+^ or CXCR3^+^IgG^+^ cells among S1^+^RBD^+^ B_SM_ in PBMCs, adenoid, and tonsils of INF and VAC participants. Samples with < 10 S1^+^RBD^+^ B_SM_ were excluded (PBMC INF N = 13, VAC N = 9; adenoid INF N = 14, VAC = 8; tonsil INF N = 22, VAC N = 6). Medians ± quartiles are shown in the box plots. For panels h and i, p values obtained from linear model correcting for participant ages (in blue) and from two-sided Mann-Whitney U test (in black) are shown. P < 0.05 was considered significant. For all panels except i, adenoid: INF N = 14, VAC N = 9; tonsil: INF N = 22, VAC N = 7, (Supplemental Table 6).

We next examined the characteristics of SARS-CoV-2-specific B cells in the pharyngeal tissues, generating 14 clusters representing naïve and unswitched memory B cells (USM), GC and pre-GC B cells (CD38^+^CD10^+^CD95^+^CD71^+^), and B_SM_ (Figure 3a–c, Extended Data Figure 2b-c, Supplemental Table 6). Like PBMCs, PCA analyses showed separation between VAC and INF subjects (Figure 3d).

We previously found that SARS-CoV-2 infection induces local GC reactions in the pharyngeal lymphoid tissues^23^. As expected, S1^+^ cells were observed in GCB clusters in most (15/24, 62.5%) INF individuals; however, two out of 10 (20%) VAC subjects also had at least 10 S1^+^RBD^+^ B cells within GCB clusters in either the tonsil or adenoid, a threshold based on a recent study^51^ (Figure 3e; Supplemental Table 7). These two VAC subjects had their most recent (second) vaccine doses 48 days and 232 days prior to surgery, suggesting this was not temporally associated with recent vaccination (Supplemental Table 2). INF tonsils had a higher proportion of S1^+^RBD^+^ B cells in the IgG^+^ GCB cluster (cluster 6) and IgA^+^ GCB cluster (cluster 11, by Mann-Whitney U) than VAC (Figure 3a–c, f–h; Extended Data Figure 2d), supporting the greater effectiveness of natural infection in generating and maintaining GC responses in pharyngeal mucosal tissues over vaccination.

Similar to PBMCs, VAC tissues had a higher frequency of S1^+^RBD^+^ B cells in certain clusters containing CD27^+^IgG^+^ B_SM_ with lower or intermediate CD21 expression and lacking CXCR3 expression. These included a greater proportion in cluster 2 in VAC tonsils, and in cluster 7 (CD62L^+^) in VAC adenoids and tonsils compared to INF (Figure 3a–c, f–h; Extended Data Figure 2c-d).

In contrast, tonsils and adenoids of INF subjects contained a higher fraction of SARS-COV-2-specific B cells in CXCR3^+^CD21^+^ containing clusters compared to VAC individuals including IgA^+^ B_SM_ populations (clusters 4 and 12) and IgG^+^ B_SM_ populations (clusters 5 and 10 in the adenoids and cluster 3 in tonsils) (Figure 3a–c, f–h, Extended Data Figure 2c-d). Increased percentages of CXCR3^+^ IgA and IgG cells in INF subjects were confirmed by manual gating (Figure 3i). CXCR3^+^ has been shown to be important for establishment of tissue-residence among memory B cells in murine lungs and production of mucosal IgA in mice, in addition to attracting cells to the mucosa^52, 53, 54^. Although we also observed CXCR3^+^ B_SM_ in the blood, we saw a trend towards higher proportions of CXCR3^+^ cells, particularly CXCR3^+^IgA^+^ cells, among S1^+^RBD^+^ B_SM_ in the adenoid and tonsil compared to blood, supporting the idea that CXCR3^+^ B_SM_ may preferentially home to mucosal tissues (Extended Data Figure 2e).

Thus, infection and vaccination were associated with phenotypically distinct memory B cell populations with a higher proportion of CXCR3^+^CD21^+^ B_SM_ including CXCR3^+^IgA^+^ cells post-infection, and B_SM_ populations with lower CD21 expression and lacking CXCR3 post-vaccination in the tissues, analogous to the peripheral blood. Furthermore, a small portion of VAC individuals showed evidence of SARS-CoV-2-specific GCBs in the pharyngeal lymphoid tissue.

### Evaluation of B_SM_ based on CXCR3 and CD21 expression

**Figure 4:**
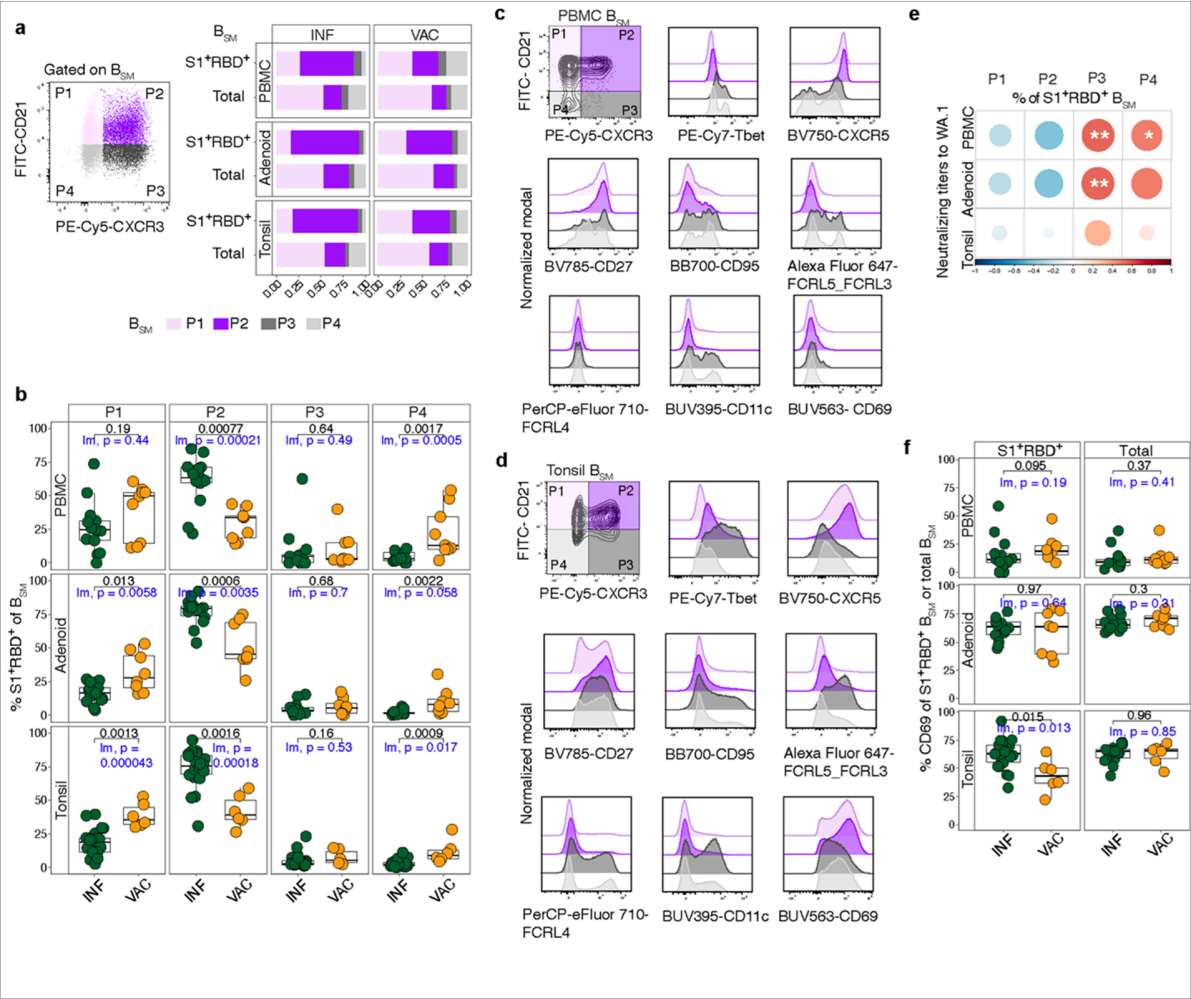
Evaluation of B_SM_ based on CXCR3 and CD21 expression (a) Left: Representative gating of B_SM_ by CXCR3 and CD21 expression yielding 4 populations (P1-P4). Right: Proportions of P1 (CXCR3^−^CD21^+^), P2 (CXCR3^+^CD21^+^), P3 (CXCR3^+^CD21^−^), and P4 (CXCR3^−^CD21^−^) among S1^+^RBD^+^ B_SM_ and total B_SM_ in PBMCs, adenoids, and tonsils of VAC and INF subjects. Samples with < 10 S1^+^RBD^+^ B_SM_ were excluded in this figure (PBMC: INF N = 13, VAC N = 9; adenoid: INF N = 14, VAC N = 8; tonsil: INF N = 22, VAC N = 6). Mean frequencies are plotted (Supplemental Table 4). (b) Comparison of percentage of P1-P4 among S1^+^RBD^+^ B_SM_ in PBMCs, adenoids, and tonsils of VAC versus INF subjects. Same samples as panel a. Medians ± quartiles are shown in the box plots. (c-d) Histogram of selected marker/antibody expression on P1-P4 subsets among B_SM_ in PBMC (c) and tonsil (d) sample. Representative sample among PBMC (N = 3) and tonsil (N = 3) are shown (Samples listed in Supplemental Table 2). (e) Correlation between percentages of P1-P4 among S1^+^RBD^+^ B_SM_ in PBMCs, adenoids and tonsils and serum neutralizing titers to WA.1. Correlations were assessed with Spearman’s rank correlation. Correlation coefficients (r values) are indicated by colors shown in the bar and size of the circle indicates absolute r value. INF and VAC groups combined for analysis (Samples in (a) with available neutralizing titers were analyzed. PBMC N = 22, adenoid N= 21, tonsil N = 27). * p < 0.05, ** p< 0.01. (f) Percentage of CD69^+^ cells among S1^+^RBD B_SM_ and total B_SM_ in PBMCs, adenoids, and tonsils of VAC versus INF subjects. Samples as those from panel a. Medians ± quartiles are shown in the box plots. In panels b and f, p values obtained from linear model correcting for participant ages (in blue) and from two-sided Mann-Whitney U test (in black) are shown in panels b and f. *P* < 0.05 were considered significant.

Given the differential expression of CXCR3 and CD21 on S1^+^RBD^+^ B_SM_ from INF and VAC individuals, we used these two markers to define 4 populations among B_SM_: P1 (CD21^+^CXCR3^−^); P2 (CD21^+^CXCR3^+^); P3 (CD21^−^CXCR3^+^); and P4 (CD21^−^CXCR3^−^) (Figure 4a). The proportion of P2 (CD21^+^CXCR3^+^) cells among S1^+^RBD^+^ B_SM_ was significantly higher in INF PBMCs and tissues compared to VAC (Figure 4b). In contrast, VAC individuals had a higher proportion of CXCR3^−^ populations, P4 in blood and tissues and P1 in tissues, highlighting differences in CXCR3 expression between antigen-specific B cells in INF and VAC subjects.

We then evaluated the expression of additional markers including transcription factors in each population in tonsil and blood. We found that atMBC markers including T-bet, CD95, CD11c, FCRL4, and FCRL3/5, were enriched primarily in P3 and P4, suggesting that these CD21^−^ populations contained the bulk of atMBCs (Figure 4c–d). Back-gating revealed that P3 and P4 contained mixtures of atMBC and acMBC (Extended Data Figure 3a). Furthermore, the frequency of P3 among S1^+^RBD^+^ B_SM_ correlated with neutralizing titers to WA.1 (Figure 4e), consistent with correlations we saw between acMBC and neutralization titers (Extended Data Figure 1h). In contrast, most cells in P2 did not express atMBC markers but expressed an intermediate-low level of T-bet, suggestive of previous exposure to IFN-g that may have driven and maintained expression of CXCR3^54, 55^. Moreover, P1 and P2, the CD21^+^ populations, expressed high levels of CXCR5, supporting a follicular origin, and were comprised primarily of cMBCs (Figure 4c–d and Extended Data Fig 3a).

CD69 is a marker of tissue resident memory B cells (B_RM_) that are poised to provide rapid local protection in response to infection or immune challenges^56^. In tonsil and adenoid B_SM_, we found that P2 had the highest frequency of CD69^+^ cells, suggesting that this population is enriched for B_RM_ (Figure 4d, Extended Data Figure 3b). Although CD69^+^ S1^+^RBD^+^ B_SM_ were noted post-infection and post-vaccination, the percentage of CD69^+^ cells among S1^+^RBD^+^ B_SM_ was significantly higher in INF tonsils compared to VAC (Figure 4f). Thus, CXCR3^+^CD21^+^ SARS-CoV-2-specific B_SM_ with B_RM_ features were enriched in upper respiratory tract lymphoid tissue after infection compared to vaccination.

### Tissue CXCR3^+^CD21^+^ S1^+^ B_SM_ have distinct transcriptomic features

**Figure 5:**
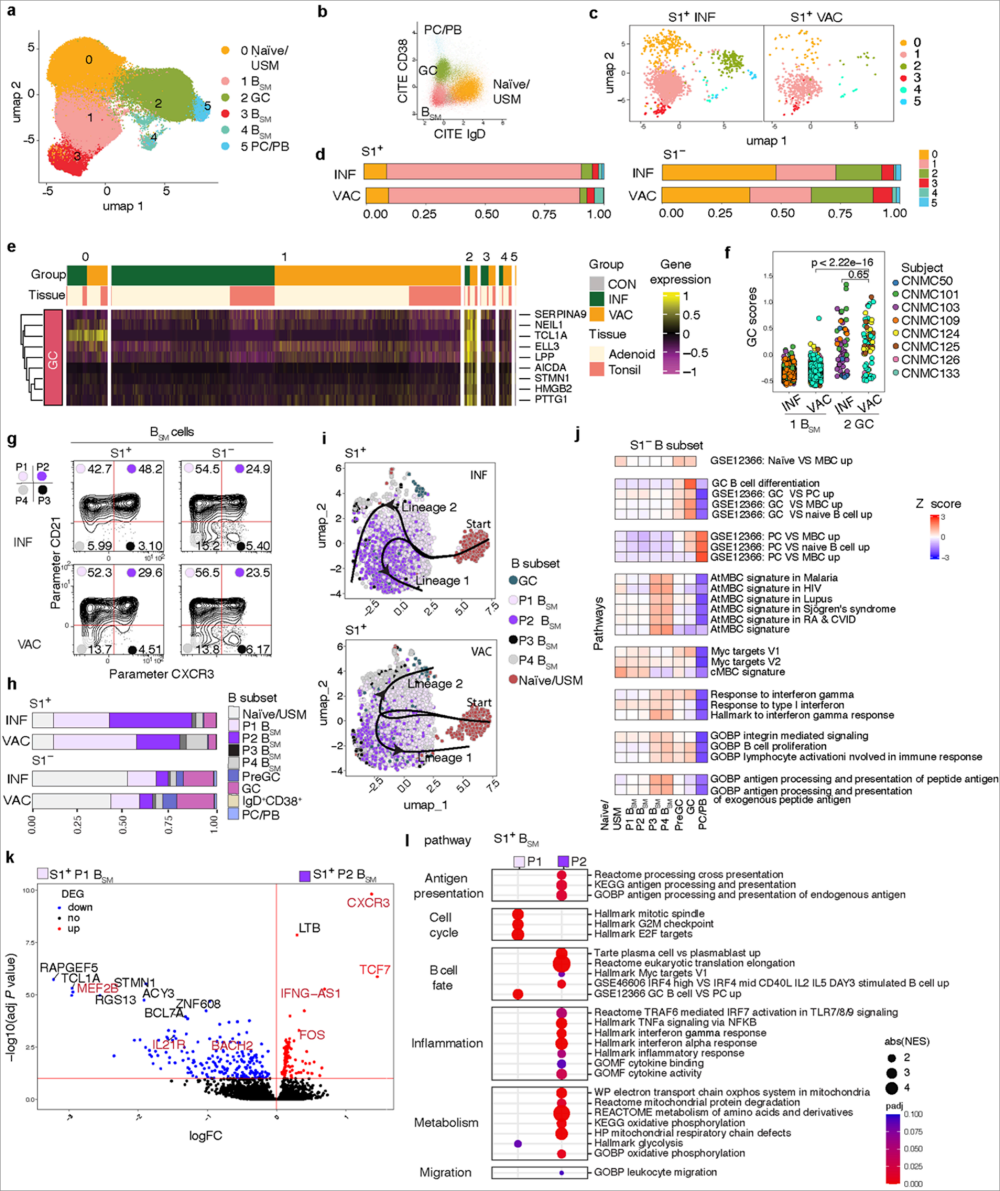
Transcriptomic features of tissue S1^+^ B_SM_ in INF and VAC individuals (a) UMAP of unsupervised clustering based on surface protein expression from CITE-seq of sorted 9370 S1^+^ and 172919 S1^−^ CD19^+^ B cells from adenoids and tonsils (adenoid: INF N = 6, VAC N = 3, CON N = 1; tonsil: INF N = 6, VAC N = 2, CON N = 2; Supplemental Table 8). (b) Expression of CD38 and IgD surface proteins by cluster. (c) Distribution of S1^+^ B cells among clusters on UMAP in INF and VAC tissues. (d) Proportion of each cluster among S1^+^ and S1^−^ B cells from INF and VAC subjects. Mean proportion among each group of subjects is plotted. (e) Heatmap showing expression of GCB signature genes among S1^+^ B cells by cluster (samples are from sample set 306–4 in Supplemental Table 8). (f) GCB signature gene module scores in S1^+^ B_SM_ and S1^+^ GCB from INF versus VAC tissues compared with two sided Mann-Whitney U test. Same samples as in panel e. (g) Representative CXCR3 and CD21 expression on concatenated S1^+^ B_SM_ and S1^−^ B_SM_ from INF and VAC tissues of CITE-seq sample set 306–4 (see Methods). (h) Proportion of each B cell subset (gated manually by surface marker expression) among S1^+^ and S1^−^ B cells of INF and VAC subjects. Same samples as in panel a. (i) Trajectory analysis of S1^+^ B cells from INF and VAC subjects using Slingshot. The naïve/unswitched memory B cell population was assigned as the starting point for trajectory construction, and B cell subsets were annotated as shown in panel h. (j) Heatmap showing gene enrichment scores for selected pathways in S1^−^ B cell subsets defined by manual gating of surface markers. Color represents z score. MBC = memory B cell (k) Differentially expressed genes (DEGs) identified by comparing pooled pseudobulk libraries of P1 versus P2 S1^+^ B_SM_ with a linear model accommodating for paired analysis. Selected genes with adjusted p value < 0.05 are labeled on the volcano plot (top 40 DEGs are listed in Supplemental Table 9). Same samples as in panel a. (l) Select pathways from gene set enrichment analysis (GSEA) of ranked genes in P2 versus P1 S1^+^ B_SM_. Dot size denotes normalized enrichment score (NES) and color denotes −log10(adjusted *p* value).

To further characterize these populations, we sorted S1^+^ and S1^−^ B cells from tonsils and/or adenoids of a subset of 8 INF, 4 VAC, and 2 CON subjects, including the two VAC subjects in whom S1^+^ GCBs were identified by ow cytometry, and performed Cellular Indexing of Transcriptomes and Epitopes by Sequencing (CITE-seq) using a panel of 20 antibodies including anti-CXCR3 and CD21. In total, 9370 S1^+^ and 172919 S1^−^ B cells were captured and assessed for surface protein expression, gene expression and BCR sequencing at the single cell level. Unsupervised clustering based on surface protein expression revealed 6 clusters representing naïve/USM, B_SM_, GCB, and plasma cells/plasmablasts (PC/PB) (Figure 5a–b). S1^+^ B cells were predominantly in a B_SM_ cluster (cluster 1) in both INF and VAC tissues (Figure 5c–d). However, a portion of S1^+^ B cells in both INF and VAC subjects were in cluster 2, which had a gene expression signature consistent with GCBs (Figure 5e–f), providing further support that spike-specific GCBs can be found in upper respiratory lymphoid tissues post-vaccination.

Comparison of surface protein to mRNA levels revealed that most CXCR3^+^ B cells had little or no detectable *CXCR3* transcripts (Extended Data Figure 4a). We, therefore, defined B_SM_ populations using CXCR3 and CD21 surface markers (P1-P4) (Figure 5g) to compare their gene expression. As expected, we saw a higher proportion of P2 among S1^+^ B cells post-infection and higher proportions of P1 and P4 (both CXCR^−^) post-vaccination (Figure 5h, Extended Data Figure 4b). Trajectory analyses using Slingshot suggested distinct patterns of S1^+^ B_SM_ development in INF and VAC conditions (Figure 5i). In INF tissues, two branches originating from naive B cells both landed at the P2 state, while in VAC tissues, two diverging trajectories from naïve B cells emerged, including one leading to the P4 population (Figure 5i).

By PCA, the CD21^+^ B_SM_ populations (P1 and P2) segregated distinctly from the CD21^−^ populations (P3 and P4) on PC1 (Extended Data Fig 4c). Both CD21^−^ populations (P3 and P4) had high expression of atMBC signature genes including *TBX21, FCRL4, NKG7, ITGAX*, and *IL2RB*^47, 57^ as well as *HCK* and *FGR*, two SRC family kinases linked to integrin signaling (Extended Data Figure 4d)^58^. P3 and P4 also had high expression of gene sets related to atMBCs, as well as antigen presentation and processing and type I and type II interferon responses, which have been previously reported to be characteristic of atMBC (Figure 5j)^39, 59, 60^.

In contrast, both CD21^+^ populations (P1 and P2) in tissues exhibited features of cMBCs including expression of *CR2* and *FCER2* and expressed high levels of the tissue-residence marker *CD69* (Figure 5j, Extended Data Figure 4d)^59^. We then evaluated differences between the CXCR3^+^ and CXCR3^−^ cMBC (P2 and P1, respectively), which constituted the majority of the S1^+^ cells. Differentially expressed genes (DEG) in P1 versus P2 S1^+^ B_SM_ revealed that cells in P2 expressed higher levels of *TCF7*, a transcription factor associated with stem-like central memory fate and found in cMBCs^36, 39^ (Figure 5k, Supplemental Table 9). Upon secondary challenge, B_SM_ in the lymphoid tissue can differentiate to PB/PC or GCBs based on their transcriptomic and epigenetic pro le^61^. DEG in P1 versus P2 S1+ B_SM_ suggested distinct cell fates upon rechallenge. P2 S1^+^ B_SM_ expressed higher *FOS*, an AP-1 transcription factor family member expressed highly in pre-plasmablasts^62^, and expressed lower levels of *BACH2*, a transcription factor that represses *BLIMP1* expression and plasma cell differentiation and is found in memory B cells that preferentially differentiate to GCBs^61^ (Figure 5k). S1^+^ P2 B_SM_ also expressed lower *IL21R*, which is critical for GCB differentiation, maintenance, and proliferation^63, 64, 65^ and the GCB marker *MEF2B* (Figure 5k)^66^. Moreover, pathway analyses revealed enrichment of genes involved PC differentiation, translation, interferon response, antigen presentation and oxidative metabolism pathways, with lower cell cycle associated genes in P2 compared to P1 S1^+^ B_SM_ (Figure 5l). Together, these differences suggest that P2 B_SM_ are better poised to differentiate to plasma cells rather than GCBs compared to P1.

We further compared the epigenetic pro les of sorted bulk P1, P2, P3 and P4 B_SM_ from three tonsils using ATAC-seq. Again, CD21^+^ (P1 and P2) and CD21^−^ (P3 and P4) populations clustered distinctly on PCA (Extended Data Figure 4e), with P3 and P4 enriched in motifs related to AP-1 transcription factors (FOS, BATF, JUN) that are associated with atMBCs and PC/PB differentiation^66^ (Extended Data Figure 4f). Direct comparison of the cMBC groups, P1 and P2, revealed that P1 had greater overall open chromatin regions compared to P2 with a few notable exceptions including the *TCF7* locus, which was more accessible in P2, consistent with the higher *TCF7* expression noted in the transcriptome (Extended Data Figure 4g, Supplemental Table 10). The enriched signaling pathways associated with differentially accessible regions in P1 versus P2 mirrored those identified in the transcriptomic analysis, including those downstream of IFN-g, BCR, and antigen receptors (Figure 5l, Extended Data Figure 4h).

*IRF4* and *PRDM1* encode transcription factors essential for PC/PB differentiation^67^; chromatin accessibility at these loci in P1 and P2 B_SM_ were similar and distinct from plasma cells and P3/P4 (Extended Data Figure 4i). However, differentially accessible regions in P2 compared with P1 had a greater proportion of AP-1-IRF composite (AICE) and interferon sequence response element (ISRE) binding motifs, which are IRF4-binding complex motifs reported to favor plasma cell differentiation. In contrast, the proportion of the Ets-IRF composite (EICE) motif, which is also a IRF4 binding complex motif but not involved in plasma cell differentiation, was comparable between P2 and P1 enriched peaks^67, 68^. This enrichment pattern suggests the P2 B_SM_ population is primed for IRF4-mediated transcriptional changes driving plasma cell differentiation. Our findings suggest that among the cMBCs, P2 B_SM_ are transcriptionally and epigenetically predisposed to antibody-secreting cell differentiation upon antigenic re-exposure compared to P1 B_SM_.

### Infection induces greater clonal expansion among S1^+^ B cells in pharyngeal tissues

To further understand differences between VAC and INF responses, we assessed isotype, somatic hypermutation, clonal expansion, and clonal overlap by BCR sequencing of the sorted S1^+^ and S1^−^ B cells characterized by CITE-seq. Single-cell BCR sequencing confirmed that the majority of S1^+^ B cells were IgG1-class switched cells in both VAC and INF tissues, with a greater proportion of IgA1 expressing cells across all B_SM_ subsets (P1-P4) post-infection (Extended Data Figure 5a).

**Figure 6:**
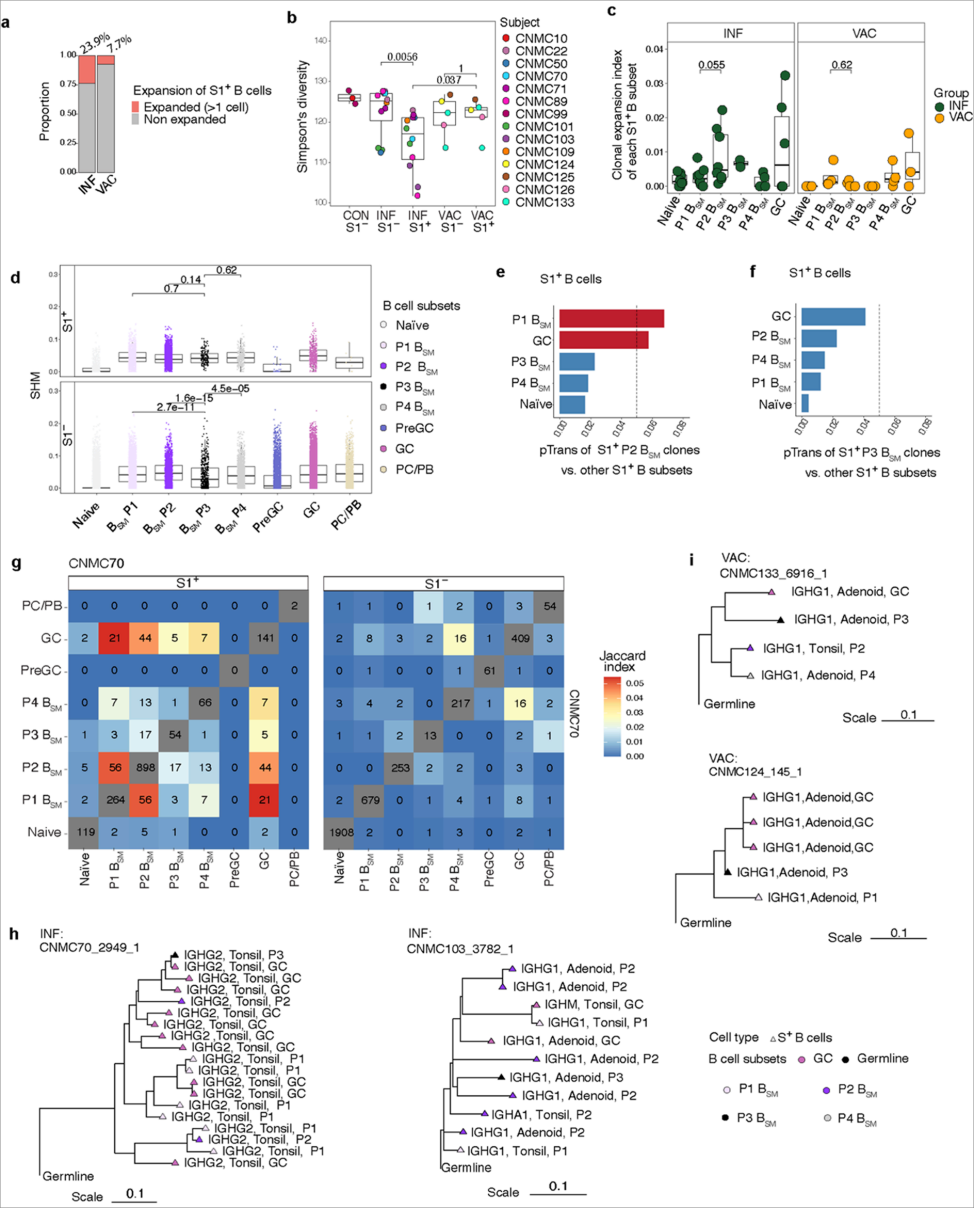
Single cell BCR sequencing of S1^+^ B cells in INF and VAC groups (a) Proportion of expanded (clone size > 1 cell) clones among S1^+^ CD19^+^ B cells from INF and VAC participants. (b) Simpson’s diversity of S1^+^ and S1^−^ B cells from adenoids and tonsils from 8 INF and 4 VAC donors and S1^−^ B cells from 2 CON. Lower Simpson’s diversity indicates a greater frequency of large clones. (c) Clonal expansion index of S1^+^ B cells in each B cell subset in INF and VAC tissues. (d) Somatic hypermutation (SHM) frequency of S1^+^ or S1^−^ B cell subsets. (e-f) Pairwise transition index (pTrans) showing degree of BCR clone overlap between P2 S1^+^ B_SM_ and S1^+^ B cell subsets (e) and between P3 S1^+^ B_SM_ and S1^+^ B cell subsets (f) from adenoids and tonsils of 8 INF and 4 VAC donors. (g) Heatmap showing clonal overlap between S1^+^ or S1^−^ B cell subsets from one representative donor who had the largest number of sorted S1^+^ B cells (CNMC 70, 2413 S1^+^ B cells). Off-diagonal elements are colored by the Jaccard index indicating degree of clonal overlap and are labeled by the raw number of overlapping clones. Diagonal elements are labeled by the total number of clones within a particular subset. (h-i) Clonal lineage trees selected from the two largest GCB bearing S1^+^ trees from INF (h) and VAC (i) donors. Triangles indicate S1^+^ cells and tip color indicates B cell subset. Isotype and tissue origin of each clone are listed next to the symbol. Branch lengths represent SHM frequency/codon in VDJ sequence according to the scale bar. Samples for a-f are listed in Supplemental Table 8; adenoid: INF N = 6, VAC N = 3, CON N = 1; tonsil: INF N = 6, VAC N = 2, CON N = 2. Significance for b-d calculated with two-sided Mann-Whitney U test. Medians ± quartiles are shown in the box plots.

A greater proportion of S1^+^ B cells were part of expanded clones in INF compared to VAC tissues (Figure 6a). S1^+^ B cells from INF tissues displayed lower clonal diversity compared to S1^−^ B cells and also to S1^+^ B cells in VAC tissues, suggesting greater antigen-driven expansion in tissues post-infection compared to post-vaccination (Figure 6b). Furthermore, in INF tissues, P2 S1^+^ B_SM_ had a trend towards a higher level of expansion than P1 S1^+^ B_SM_; this expansion was not seen in P2 S1^+^ B_SM_ post-vaccination (Figure 6c).

In tonsil, atMBCs have been noted to have slightly lower levels of SHM^59^; we also found that SHM frequency was lower among bulk S1^−^ P3 B_SM_ in the tissues. However, SHM frequency was comparable across all four P1-P4 subsets of S1^+^ B_SM_ in both INF and VAC (Figure 6d), revealing distinctions between SARS-CoV-2-specific cells compared to the bulk. Indeed, SHM frequency and heavy chain CDR3 amino acid length and distribution among S1^+^ IgG^+^ B_SM_ and GCBs were comparable between INF versus VAC tissues (Extended Data Figure 5b-d).

We then assessed BCR clonal overlap to evaluate B_SM_ development. Using STARTRAC (single T cell analysis by RNA sequencing and TCR tracking) pairwise transition index (pTrans)^69, 70^, which assesses developmental links in lymphocyte populations using clonal sharing, we found that antigen-specific S1^+^ P2 B_SM_ exhibited high pTrans scores with S1^+^ P1 B_SM_ and S1^+^ GCB (Figure 6e). Although S1^+^ P3 B_SM_ were most strongly connected to GCBs, their connectivity was less strong than S1^+^ P2 B_SM_ (Figure 6f). Bulk S1^−^ P2 B_SM_ also had a strong association to S1^−^ GCBs, whereas S1^−^ P3 B_SM_ were most strongly associated with S1^−^ P4 B_SM_ and PC/PB, further supporting distinct connections and development among these cell populations (Extended Data Figure 5e-f).

We then looked further at BCR sequences from the tissues of one INF subject from whom we sorted a large number of S1^+^ B cells (CNMC 70). By Jaccard index scores, we observed clonal overlap among S1^+^ P1, P2, P3, and P4 B_SM_ populations, with particularly strong overlap between P1 and P2. S1^+^ P1 and P2 B_SM_ also had strong clonal sharing with GCBs, whereas S1^+^ P3 and P4 B_SM_ had GC overlap but to a lower extent than S1^+^ P1 and P2 B_SM_ (Figure 6g). Evaluation of clonal trees showed S1^+^ B_SM_ bearing various phenotypes (P1-P4, GCB) and isotypes emerging from a common ancestor in both INF (Figure 6h) and VAC (Figure 6i) tissues, implying broad differentiation potential or plasticity in B cell phenotypes during clonal expansion. Several clonotypes were also shared among adenoid and tonsil S1^+^ B cells, particularly in P1 and P2 B_SM_ and GCB populations, supporting an immunologic connection between these two oropharyngeal lymphoid tissues, as we previously reported^23^ (Figure 6h–i, Extended Data Figure 5g). Thus, although similar levels of SHM were noted post-infection and vaccination, infection induced greater antigen-specific B cell expansion in the tissues, particularly among P2 B_SM_, which likely have a GC origin.

### CXCR3^+^CD21^+^ B_SM_ are more responsive to BCR stimulation and poised to differentiate to plasmablasts

**Figure 7:**
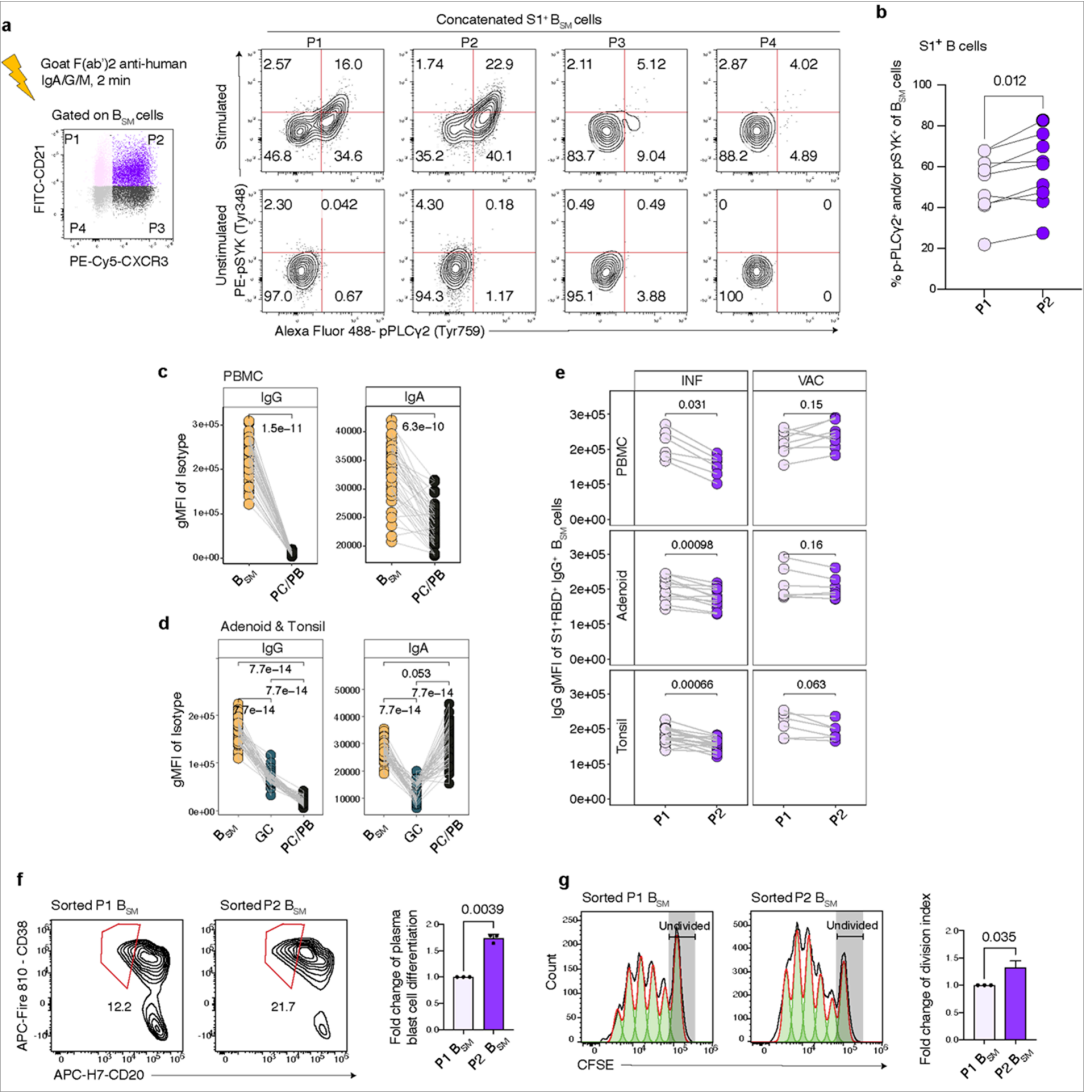
CXCR3^+^CD21^+^ (P2) B_SM_ are predisposed to plasma cell differentiation (a) Phosphorylation of Syk and PLCg2 in S1+ B_SM_ subsets, concatenated by group, with and without 2-minute stimulation with soluble anti-IgG, -IgA, and -IgM. Representative plots are shown (N = 10 tonsils). (b) Percentage of cells expressing either or both p-Syk and/or p-PLCg2 among P1 versus P2 S1^+^ B_SM_ post-BCR stimulation (percentages of p-Syk and/or p-PLCg2 from untreated conditions were subtracted from stimulated conditions). Same samples as in panel a. (c-d) Geometric mean fluorescence intensity (gMFI) of surface IgG or IgA on their respective isotype-specific B cell subsets from PBMC (c) and tonsil/adenoid (d) (PBMC N = 37, adenoid N = 33, tonsil N = 41). (e) gMFI of IgG on P1 versus P2 S1^+^RBD^+^ IgG^+^ B_SM_ in INF and VAC groups. Samples with at least 10 cells in both P1 and P2 S1^+^RBD^+^ IgG^+^ B_SM_ populations were analyzed (PBMC: INF N = 6, VAC N = 8; adenoid: INF N = 12, VAC N = 7; tonsil: INF N = 17, VAC N = 6). (f) Percentages of plasmablasts (CFSE^+^CD38^+^CD20^−^) after 4 days of *in vitro* culture of sorted P1 and P2 B_SM_ from tonsils with B cell depleted PBMCs from an unrelated donor, R848, and IL-2. Fold change comparing P1 and P2 was calculated from paired P1 versus P2 B_SM_ from three tonsils. (g) Division index of CFSE-labelled P1 and P2 B_SM_ following *in vitro* culture described in Figure 7f. P values for panels b-g were calculated with two-sided Wilcoxon signed ranks test (paired). Samples used in this figure are listed in Supplemental Table 2. In f and g, mean ± SD are shown in the box plots.

To determine whether these differences reflect functional differences among P1-P4 B_SM_ populations, we evaluated responsiveness to BCR stimulation, proliferation capacity, and ability to differentiate to antibody-secreting cells. Tonsil cells from a separate group of pediatric subjects who were either post-infection or had hybrid immunity to SARS-CoV-2 (both infected and vaccinated, Supplemental Table 2) were stimulated with soluble anti-human IgA, IgG, and IgM for 2 minutes and phosphorylation of the tyrosine kinase Syk and phospholipase Cg2 (PLC-g2), two downstream signaling proteins rapidly phosphorylated upon BCR stimulation, were measured^71^. As noted previously, the CD21^lo^ B_SM_ populations (P3 and P4) were poorly responsive to BCR stimulation with soluble immunoglobulin^72, 73^ (Figure 7a). In contrast, the P2 B_SM_ population had the strongest induction of p-Syk and/or p-PLCg2 among total B_SM_ and S1^+^ B_SM_ (Figure 7a–b, Extended Data Figure 6a-b). This phenotype was not limited to B_SM_, as the CXCR3^+^CD21^+^ (P2) subpopulation also had the strongest response to BCR stimulation among GCBs (Extended Data Figure 6c).

In general, as B cells transition to PC/PB, membrane-associated IgG declines due to altered splicing, generating secreted IgG^36, 74^. We analyzed surface IgG and IgA expression (geometric mean fluorescence intensity or gMFI of anti-IgG and anti-IgA antibody staining) among B_SM_ and PC/PB in our ow cytometry data. As expected, CD27^hi^CD38^hi^ PC expressed marked reductions in surface IgG compared to B_SM_ in both the peripheral blood and tissues (Figure 7c). Surface IgA was also reduced on plasma cells in peripheral blood, but to a lesser extent (Figure 7c) and was not reduced on tissue PC compared to GCBs and tissue B_SM_ (Figure 7d). These observations are consistent with findings of functional surface IgA on gut and bone marrow PC in humans^75^ and suggest that surface IgA may also play a functional role on PC in the oropharyngeal lymphoid tissue.

We then compared surface IgG expression on the two major S1^+^RBD^+^ B_SM_ populations, P1 and P2, as a surrogate for PC differentiation^36^. In INF tissues and blood, S1^+^RBD^+^ IgG^+^ P2 B_SM_ had lower surface immunoglobulin expression than their P1 counterparts, suggesting the SARS-CoV-2-specific P2 B_SM_ post-infection are more prone to differentiate to PC (Figure 7e). Significant differences were not noted for P1 and P2 in VAC tissues, although a trend was seen in the tonsils (Figure 7e). Moreover, in the tonsil, S1^+^RBD^+^ IgG^+^ B_SM_ from INF subjects had lower surface immunoglobulin expression compared to VAC subjects (Extended Data Figure 6d); a similar trend was noted in the adenoid, suggestive of a propensity of tissue B_SM_ induced by infection to differentiate to PC/PB.

We then directly assessed the ability of P1 and P2 B_SM_ from tissues to differentiate to plasmablasts and proliferate *in vitro*. We sorted bulk P1 and P2 B_SM_ cells from tonsils of three subjects with hybrid immunity and labelled them with carboxyfluorescein succinimidyl ester (CFSE). We cultured the labelled P1 and P2 B_SM_ populations with B cell-depleted PBMCs of an unrelated donor, R848 (TLR7/8 agonist), and IL-2 and we assessed proliferation index and percentage of plasmablasts in culture using ow cytometry after 4 days. Due to low cell numbers and poor viability after sorting, we were unable to reproducibly culture and assess P3 and P4 B_SM_. Compared to P1, a greater proportion of P2 B_SM_ differentiated to CD38^+^CD20^lo^ plasmablasts (Figure 7f). P2 B_SM_ also exhibited a higher proliferation index in culture (Figure 7g). Thus, P2 B_SM_, which were enriched among INF samples, were better primed for plasma cell differentiation compared to P1 B_SM_, which may result in greater protective antibody production in the upper respiratory tract tissues upon re-exposure to SARS-CoV-2 in infected individuals compared to vaccinated individuals.

### Infection, but not vaccination, is associated with persistent immunologic activation in pharyngeal tissues

**Figure 8:**
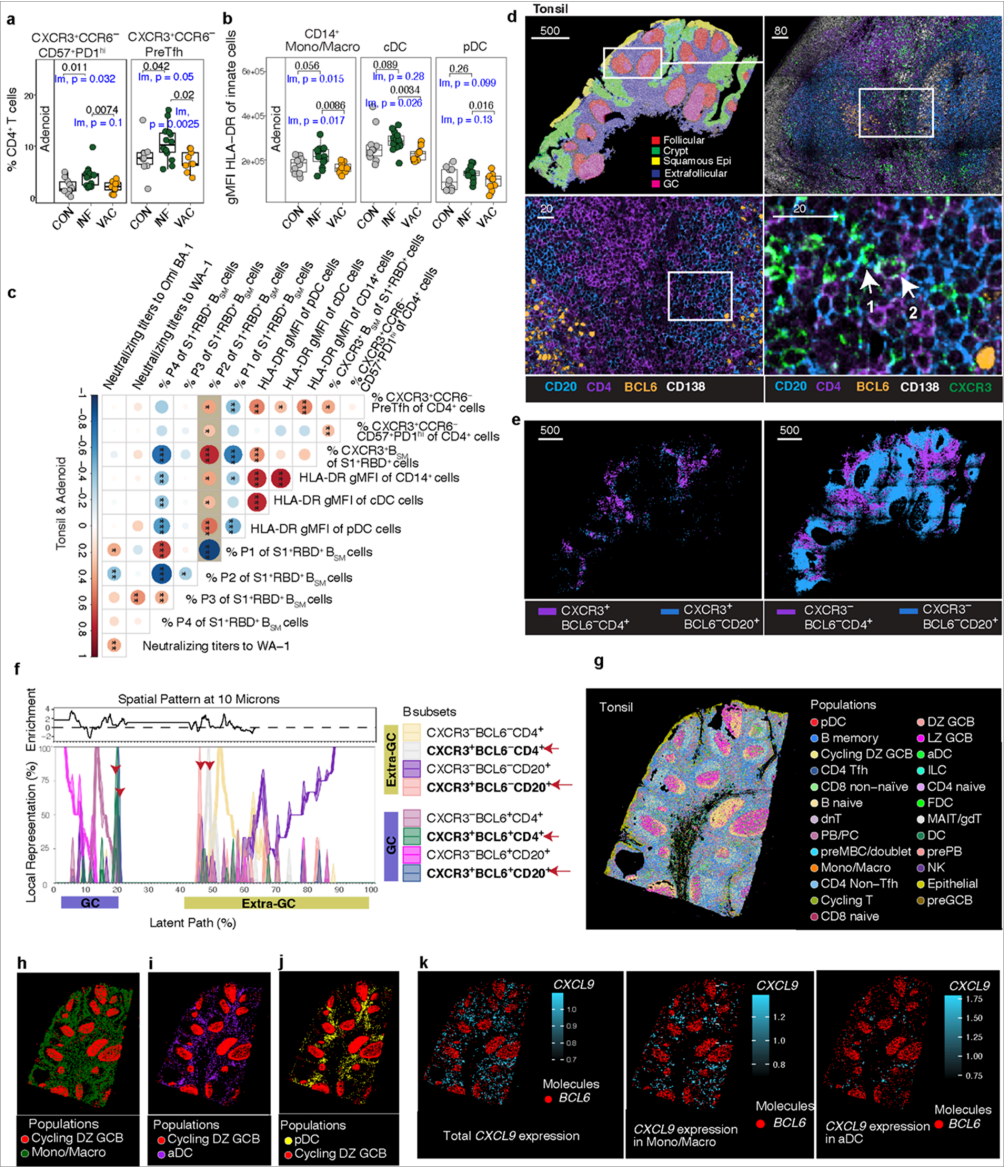
CXCR3^+^ cells and CXCL9-expressing cells co-localize in the tissue (a) Percentages of CXCR3^+^CCR6^−^CD57^+^PD1^hi^ and CXCR3^+^CCR6^−^ pre-Tfh among CD4^+^ T cells in CON (N = 10), INF (N = 14) and VAC (N = 9) adenoids. Samples listed in Supplemental Table 2. (b) gMFI of HLA-DR expression on CD14^+^ monocytes, conventional dendritic cells (cDC) and plasmacytoid dendritic cells (pDC) in CON, INF and VAC adenoids. Same samples as in panel a. For panels a and b, p values obtained from linear model correcting for participant ages (in blue) and from two-sided Mann-Whitney U (in black) are shown. *P* < 0.05 were considered significant. Medians ± quartiles are shown in the box plots. Comparisons of VAC versus CON were not statistically significant in both panels (not shown). (c) Correlations between neutralizing titers to omicron BA.1 and WA-1 strains, percentages of P1-P4 among S1^+^RBD^+^ B_SM_, CXCR3^+^CCR6^−^CD57^+^PD1^hi^ among CD4^+^ T cells, and CXCR3^+^CCR6^−^ preTfh among CD4^+^ T cells and HLA-DR gMFI of pDC, cDC, and CD14^+^ monocytes. Data from tonsils and adenoids and INF and VAC were pooled (N = 49). Correlations were assessed with Spearman’s rank correlation. Correlation coefficients (r values) are indicated by color shown in the bar. Size of the circle indicates absolute r values. Significant p values are indicated as * p<0.05, ** p<0.01, *** p<0.001. (d) Immunofluorescence images of tonsil from one INF donor, representative of paired tonsil and adenoid from one INF and one VAC donor, with major histologic regions delineated (top left). Insets show greater magnifications. Bottom panels show CD20 (blue), CD4 (purple), BCL6 (yellow), and CD138 (plasma cells and epithelial cells, white) with and without CXCR3 channel (green) shown. Arrows indicate CXCR3^+^CD20^+^ B cells (arrow 1) and CXCR3^+^CD4^+^ T cells (arrow 2). (e) Images showing the localization of CXCR3+BCL6^−^ CD4^+^ T and CD20^+^ B cells (left panel) and CXCR3−BCL6^−^ CD4^+^ T and CD20^+^ B cells (right panel) in the representative tonsil from panel d. (f) Spatial distribution of 8 cell subsets defined by CD4, CD20, BCL6, and CXCR3 expression, shown in the representative tonsil from (d) using SPACE at 10 mm scale. Peaks of local representation of each population along the latent path indicate their spatial proximity to each other. BCL6^+^ and BCL6^−^ cells were gated with a gap in between, which is reflected as a gap in the latent path. (g) Annotated cell types based on transcriptome are shown in nearby section of the representative tonsil from panel d run on Xenium In Situ spatial transcriptomic platform using human 5000 gene panel. 521,787,433 transcripts and 1,834,484 cells were detected after quality filtering. (h-j) Spatial map showing the location of monocytes/macrophages (Mono/Macro) (h), activated dendritic cells (aDC) (i), pDC (j) and cycling dark zone GCB (DZ GCB) to indicate germinal centers from the representative tonsil from panel d. (k) Spatial map showing the location and expression levels of CXCL9, both overall and specifically within mono/macro and aDC cells, in the representative tonsil from (d).

We previously found that SARS-CoV-2 infection early in the pandemic induced persistent expansion of CD4^+^ and CD8^+^ T cell populations associated with antiviral, GC and IFN-g responses in the tonsils and adenoids compared to uninfected controls, with the most significant effects in adenoid^23^. However, whether vaccination also induces long-lasting immunologic changes in the upper respiratory tract lymphoid tissues is unclear. Consistent with our previous findings, a higher proportion of CXCR3^+^CCR6CD57^+^PD-1^hi^ CD4^+^ T cells and CXCR3^+^CCR6^−^ pre-Tfh cells were noted in INF compared to CON adenoids (Figure 8a; Extended Data Figure 7a). In contrast, VAC adenoids did not exhibit changes in these T cell populations compared to CON (Figure 8a).

COVID-19 induces persistent activation and epigenetic remodeling of innate immune cells including peripheral blood monocytes and dendritic cells well into convalescence^76, 77^. The frequency of CD14^+^ monocytes/macrophages, conventional dendritic cells (cDC), and plasmacytoid dendritic cells (pDC) did not differ among the VAC, INF, and CON tissues (Extended Data Figure 7b). However, these innate immune populations expressed higher HLA-DR in INF adenoids compared to VAC (and trended toward higher expression compared to CON), revealing persistent myeloid cell activation in adenoids post-infection but not post-vaccination (Figure 8b). Unlike the tissues, peripheral blood CD14^+^ monocytes/macrophages and cDC in INF subjects did not express higher HLA-DR post-infection, highlighting localized differences in the impact of infection in upper respiratory tract tissues compared to blood (Extended Data Figure 7c). However, the antiviral type I interferon-producing and CXCR3-expressing pDC population was slightly more activated in both INF blood and tissues (Figure 8b, Extended Data Figure 7c)^78^. Thus, convalescence was associated with local activation of innate cells in tissues, which may reflect differences in the primary lymphoid site of antigen-presentation and GC response and/or antigen persistence in infection versus vaccination.

We then assessed correlations between populations of interest. The proportion of P2 cells among S1^+^RBD^+^ B_SM_ was positively associated with the proportion of CXCR3^+^CCR6^−^ pre-Tfh cells, an IFN-g producing population that is involved in GC reactions and important for CXCR3^+^ B_RM_ development in mice^23, 54^ (Figure 8c). The frequency of the CXCR3^+^CCR6^−^ CD57^+^PD-1^hi^ Tfh cell population also significantly correlated with the percentage of P2 S1^+^RBD^+^ B_SM_ (Figure 8c): these CXCR3^+^ Tfh populations also correlated with CXCR3^+^ total and/or S1^+^RBD^+^ B_SM_ (Extended Data Figure 7d). Moreover, the proportion of P2 B_SM_ and CXCR3^+^ pre-Tfh cells were also positively associated with the activation levels of monocytes/macrophages, cDC, and pDC, pointing to the potential relevance of innate cell activation in development or accumulation of CXCR3^+^ lymphocytes (Figure 8c). Thus, infection is associated with prolonged activation of innate or expansion of adaptive immune populations in tonsils/adenoids, which may contribute to the generation of CXCR3^+^ B_SM_.

### CXCR3^+^ lymphocytes and CXCL9-expressing myeloid cells are in close proximity in the interfollicular region

Using multicolor immunofluorescence imaging, we compared the location of CXCR3^+^ CD20^+^ B and CD4^+^ T cells and to their CXCR3^−^ counterparts in 4 tissue samples, derived from tonsils and adenoids of one INF and one VAC subject (Figure 8d, Supplemental Table 2). CXCR3^+^BCL6^−^ CD20^+^ B cells and CXCR3^+^BCL6^−^ CD4^+^ T cells were primarily located in the extrafollicular region (Figure 8d, Extended Data Figure 7e-f). Compared to their CXCR3^−^ counterparts, a smaller portion of CXCR3^+^ BCL6^−^ B cells were in follicles, which includes the GC and surrounding mantle (Extended Data Figure 7e). A similar pattern of distribution was seen among CXCR3^+^ CD4^+^ T cells (Extended Data Figure 7f). CXCR3^+^BCL6^−^ CD4^+^ T cells and CXCR3^+^BCL6^−^ CD20^+^ B cells appeared co-localized or aggregated near one another compared to CXCR3^−^ BCL6^−^ CD4^+^ T and CXCR3^−^BCL6^−^ CD20^+^ B cells which were more diffusely distributed in the extrafollicular area (Figure 8d and e).

We then assessed spatial proximity of CXCR3^+^ and CXCR3^−^ B and CD4^+^ T cells using spatial patterning analysis of cellular ensembles (SPACE), which assesses spatial relationships among multiple cell populations^79^. Peak areas of abundance of BCL6^+^ and BCL6^−^ populations were distinct, clearly delineating GCs (Figure 8f). Within BCL6^−^ regions, the CXCR3^+^BCL6^−^CD20^+^ B cell and CXCR3^+^BCL6^−^CD4^+^ T cell peaks were in close proximity to each other; similarly, in BCL6^+^ regions, which represent GCs, the CXCR3^+^ BCL6^+^ CD20^+^ B cell and CXCR3^+^ BCL6^+^ CD4^+^ T cell were closely aligned, suggesting that CXCR3^+^ B and T cells were adjacent both within and outside GCs (Figure 8f). Moreover, compared to their CXCR3^−^ counterparts, CXCR3^+^BCL6^−^ B and T cells were closer to BCL6^+^ populations, and in particular, closer to CXCR3^+^BCL6^+^ B and T cells, further implying proximity and possible interactions among CXCR3^+^ Tfh, GCB, and B_SM_ populations in the GC and T-B border regions (Figure 8f).

We performed spatial transcriptomics analysis with the Xenium 5000 human gene panel of the same 4 samples from an adjacent section of tissue. With unsupervised clustering and annotation, we identified 25 cell types, which localized to particular regions (Figure 8g, Extended Data Figure 7g). CXCL9, −10, and −11 are ligands of CXCR3 and enable migration and retention of CXCR3-expressing lymphocytes in tissue^80^. In tonsils and adenoids, we found that macrophages/monocytes and dendritic cells were the primary expressors of *CXCL9, CXCL10*, and *CXCL11*, with *CXCL9* being the most abundantly expressed (Extended Data Figure 7h). Moreover, these innate immune cell populations were located in the interfollicular area, where we detected expression of *CXCL9* and where we found CXCR3^+^ B and T cells by immunofluorescence (Figure 8d, g–k). Thus, spatial analyses suggest that the CXCR3 and CXCL9/10/11 axis facilitates interaction of innate and adaptive immune cells in lymphoid tissue, which may shape both the position and characteristics of memory B cells during viral infection.

## DISCUSSION

Maintenance of virus-specific adaptive immune memory in the respiratory mucosa is important for immune protection against respiratory viral infections including COVID-19. By assessing tonsils and adenoids of children undergoing tonsillectomy/adenoidectomy, we found SARS-CoV-2-specific B cells, including B_SM_, B_RM_ and a few GCBs, in these upper respiratory tract lymphoid tissues following vaccination. Distribution of B_SM_ across tissues, including mucosal tissues, after intramuscular vaccination has been noted^51, 81, 82^. However, whether and how these cells differ from memory cells found post-infection has not been clear. Using high-dimensional analyses, we show that immune memory is maintained in mucosal lymphoid tissues after intramuscular COVID-19 immunization, but reveal distinct features compared to those generated post-infection. In particular, we identified a subset of cMBC defined by CXCR3^+^CD21^+^, that was enriched in infected individuals, with increased propensity for plasma cell differentiation and distinct localization within mucosal lymphoid tissues. This population may be responsible for some of the qualitative and clinical differences in humoral mucosal immunity observed with infection and vaccination.

Prior studies have found SARS-CoV-2-spec c GC and Tfh cells in the draining axillary lymph nodes following mRNA vaccination^17, 18, 19, 20, 83^, but no SARS-CoV-2-specific GC B cells were noted in a small number of contralateral lymph nodes^17^. However, spike-reactive GCBs have been noted in lung-associated lymph nodes of a few (2/18) vaccinated (and uninfected) organ donors^51^ and tonsils of a few vaccinated adults, although whether these latter subjects were truly SARS-CoV-2 infection-naïve is unclear since some had B cells reactive to NC^60^. To address this concern, we measured both anti-NC antibodies and anti-ORF8 antibodies, the latter of which are more sensitive than antibodies to NC^24^. We too noted the presence of spike-specific GCBs in some vaccinated participants in distal mucosal lymphoid sites, although fewer than among infected subjects. Whether these result directly in response to immunization, from cross-reactivity to common cold coronaviruses, or exposure to SARS-CoV-2 that did not result in a productive infection, perhaps due to the presence of high affinity antibodies generated by vaccination^84, 85^, remains an interesting question.

Although COVID-19 mRNA vaccines have high vaccine efficacy and prevent hospitalization and death from COVID-19, epidemiologic studies suggest that infection provides longer-lasting immunity than vaccination^6, 7, 8^. This may result from differences in the route of viral antigen exposure to the host’s immune system (intramuscular vs. inhaled), breadth of antigenic exposure (spike only vs. all viral antigens), nature of antigens presented (lipid nanoparticles vs. virions) or duration of antigen exposure. These factors, in turn, affect host immune responses, and, indeed, we found that infection and vaccination drive the generation of phenotypically different SARS-CoV-2-specific B cells, suggesting unique B_SM_ developmental pathways depending on the type of exposure.

The clonal expansion and persistence of CXCR3^+^ B_SM_ post-infection suggest that IFN-g, induced by viral infection and likely produced by activated and expanded innate and Tfh cells in the tissue, may be a driving force for these developmental differences in B cell memory. The CXCR3^+^ P2 B_SM_ population expressed low levels of Tbet, which may reflect prior IFN-g exposure during infection, and were distinct from atMBCs which expressed high levels of Tbet. Our results suggest that CXCR3 expression affords two key characteristics incMBCs that may contribute to enhanced mucosal protection post-infection. First, CXCR3 promotes homing and retention of B_SM_ cells, particularly IgA^+^ cells, to mucosal lymphoid tissues, where CXCR3^+^ cells bear B_RM_ markers, as supported by murine studies^52, 54^. Second, we found that among cMBCs, CXCR3^+^CD21^+^ B_SM_ have a predilection for plasma cell differentiation upon secondary challenge compared to CXCR3^−^CD21^+^ B_SM,_ enabling a more robust antibody response upon secondary challenge. Although CXCR3^+^ B_SM_ were enriched in the tissues, the difference in CXCR3 expression between INF and VAC was also clear in PBMCs suggesting CXCR3 may be a useful marker for evaluating potential for trafficking even in blood; this is supported by our recent findings in blood of vaccinated adults with hybrid immunity^86^, indicating the applicability of our findings across age groups. Moreover, although our study focused on mRNA COVID-19 vaccines, another recent study found the Ad26.COV2.S, elicited more CXCR3^+^ B_SM_ without atMBC features compared to mRNA vaccines^5^, suggesting similar cells may be generated by viral vaccine platforms. Nonetheless, the mRNA component of mRNA-lipid nanoparticle vaccines can stimulate IFN-g production by Tfh cells^87^, potentially explaining the presence of some CXCR3^+^ B_SM_ in the blood and tissues of vaccinated subjects in our study. Of note, the frequency of the P2 population among B_SM_ did not correlate with serum neutralizing titers, suggesting this population reflects other aspects of immunity induced by infection and that neutralizing titers cannot be used as a proxy for this population.

Conversely, we found that mRNA vaccination induced a greater proportion of virus-specific CD21^lo^ and particularly CXCR3^−^ B_SM_ populations than infection in both blood and tissues^29, 38, 60^. Although some studies suggest that CD21^lo^ atMBCs have lower degrees of somatic hypermutation than cMBC and emerged from extrafollicular reactions particularly in the context of chronic viral or autoimmune conditions^39, 45^, we found that SARS-CoV-2-specific CD21^lo^ populations in tonsils/adenoids had comparable levels of SHM as CD21^+^ B_SM_, highlighting the importance of assessing antigen-specific versus bulk cell populations and evaluating cells in patients without conditions characterized by strong extrafollicular reactions. The frequency of CD21^− /lo^ B_SM_ in the blood declines with time after influenza or COVID-19 vaccination, suggesting these cells may be shorter lived^34, 35^. It is possible that the enrichment of SARS-CoV-2-specific CD21^−^ cellspost-vaccination accounts for the steeper decline in immunity months after mRNA vaccination compared to infection. Nonetheless, FCRL5^+^Tbet^+^ memory B cells with atMBC features have been found 1 year after influenza vaccination and contributed significantly to serum antibody levels upon secondary challenge; similarly FCRL4^lo^ atMBCs were present at least 6 months post-influenza vaccination, suggesting the picture is more complex^36, 37^. We also note that vaccination engendered broader variant coverage, as determined by cross-reactivity to omicron, highlighting an important feature induced by mRNA vaccination. Thus, the plasticity and function of different memory populations following vaccination versus infection remain important questions.

As humans are confronted with new pathogens, the development of vaccinations for respiratory pathogens that elicit strong mucosal immunity, which may reduce mucosal shedding and transmission, is a priority. Although intramuscular SARS-CoV-2 mRNA vaccines generate rapid and broad immunity, they elicit low neutralizing antibody levels in the respiratory tract^30, 31^. Trials of intranasal vaccines in humans are ongoing and have shown mixed results^88, 89^. Nonetheless, pre-clinical studies of inhaled vaccine boosters following intramuscular mRNA vaccines in murine models showed enhanced mucosal protection due to trafficking of antigen-experienced B_SM_ to the respiratory tract through the CXCL9/10-CXCR3 axis and enhanced differentiation to IgA-producing plasma cells^48^. Our study underscores the significance of this axis in homing and shaping immunity in the upper respiratory tract of humans. In summary, our study provides evidence for the generation and maintenance of mucosal B cell memory after mRNA vaccination and further provides a framework for evaluating immunity in the upper respiratory tract and the blood following immunization.

## LIMITATIONS

Although study participants were heterogeneous in terms of their age, tonsil condition, time since vaccination/infection, and doses of immunization, we tried to control for age as a covariate using a linear model. Moreover, the INF cohort, which was collected in 2020–2021, had similar times from their last known immunologic exposure to surgery as the VAC cohort and were exposed to early circulating strains with spike proteins similar to the strain in the first-generation mRNA vaccines, helping control for these factors. Nonetheless, we only know the time of infection for approximately half of the infected subjects. As our study is a cross-sectional study, we are unable to assess temporal changes in immune responses over time. We also note that our INF cohort only included patients with mild or asymptomatic infection; severe infection may alter or compromise the humoral immunity generated by infection^90^. Lastly, due to the increasing percentage of infected individuals, our sample size of vaccinated only individuals was limited, which could affect our ability to fully assess correlations between clinical characteristics and immune profiles.

## METHODS

### Participant recruitment

This study was approved by the Institutional Review Board (IRB) at Children’s National Hospital (IRB protocol number 00009806). Written informed consent was obtained from parent/guardians of all enrolled participants, and assent was obtained from minor participants over 7 years of age.

We recruited 21 children who underwent tonsillectomy and/or adenoidectomy at Children’s National Hospital (CNH) in Washington, DC, USA and also received at least one dose of a first generation COVID-19 mRNA vaccine. The first 17 participants were recruited from December 2021 to April 2022, and the remaining were recruited from August 2022 to September 2022. Because not all tissues or blood were available from each subject, we collected a total of 21 blood samples, 19 adenoids, and 18 tonsils from these 21 participants. No statistical methods were used to predetermine sample size. All participants had negative RT-PCR testing from a nasopharyngeal swab for SARS-CoV-2 within 72 hours of surgery. Demographic information and clinical data were collected through parental questionnaires and chart review and inputted and managed in REDCap (https://project-redcap.org/), and biologic samples were acquired in the operating room by the clinical team at CNH. Participants with no evidence of prior infection (negative for anti-nucleocapsid (NC) or anti-open reading frame 8 (ORF8) serum antibodies and/or no clinical history of PCR/antigen confirmed SARS-CoV-2 infection) were classified as the VAC group (vaccinated only, 10 subjects). Samples from the remaining 11 subjects with hybrid immunity (both post-vaccinated and infection, 11 subjects) and additional post-infection subjects without vaccination who were recruited in 2022/2023 (12 subjects) were used in some assays.

We also used samples obtained from our prior study of 24 children infected with SARS-CoV-2 from 2020 to 2021 (INF), prior to the availability of vaccines for children. Collection of these samples was previously described.^23^ Prior infection in these individuals was confirmed by serologic testing for anti-spike antibodies and/or the presence of SARS-COV-2-specifc B cells in the blood, tonsil, or adenoid by ow cytometry. From these 24 children, 15 PBMCs, 14 adenoids, and 22 tonsils were analyzed based on availability of cells (Supplementary Table 2).

### Blood and tissue collection

Blood samples were obtained just prior to the surgical procedure in the operating room in serum separator tubes (BD) for serum collection and sodium heparin tubes (BD) for peripheral blood mononuclear cells (PBMCs) extraction from an intravenous line placed for anesthesia. Once received in the laboratory on the day of collection, serum separator tubes were spun at 1200 g for 10 min, and serum was aliquoted and stored at −80°C. PBMCs were isolated the day after collection by density gradient centrifugation (Lymphocyte Separation Medium, MP Biomedicals) at 1500 rpm for 30 min at room temperature (RT) with no brake and washed with PBS. If red blood cell contamination was present, cells were lysed with ammonium-chloride-potassium (ACK) buffer (Gibco).

Tonsil and adenoid tissues were stored in RPMI-1640 (RPMI) media with 5% heat-inactivated fetal bovine serum (FBS, VWR), gentamicin 50 mg/mL (Gibco), and 1 × antibiotic/antimycotic solution (Gibco) on ice immediately after collection. Tissues were processed the day after collection. The tissue was mechanically disrupted and filtered through a 100 μm cell strainer to create a single cell suspension, lysed with ACK buffer, and washed with PBS three times. Cells were then stored in liquid nitrogen in the presence of FBS with 10% DMSO and thawed according to our published protocol^92^ for ow cytometry, CITE-seq, and functional assays below.

### Seroreactivity of samples to SARS-CoV-2 spike, nucleocapsid and ORF8 by ELISA

96 well Immulon plates were coated with 20 ng/100 μL of recombinant nucleocapsid, spike RBD protein, or Open Reading Frame 8 (ORF8) from WA1/2020 in PBS overnight at 4 °C. Starting at a 1:20 dilution, serum samples were serially diluted 5-fold and applied to the coated well for 1 hr at ambient temperature. Serum samples were assayed in duplicate, as described before^13, 93^. After three washes with PBS/0.05% Tween 20, bound human IgG antibodies were detected with 1:5000 dilution of HRP-conjugated anti-human IgG Fc-specific antibody (Jackson Immuno Research). After 1 hr, plates were washed with PBST followed by PBS, and o-Phenylenediamine dihydrochloride (OPD) was added for 10 min. Absorbance was measured at 492 nm. End-point titer was determined as 3-fold above the average of the absorbance values of the binding of serum samples to blank control wells. The end-point titer is reported as the serum dilution that was above this cutoff and was calculated using Prism 9 (GraphPad Software).

### SARS-CoV-2 serum neutralization assay

Samples were evaluated in a quali ed SARS-CoV-2 pseudovirion neutralization assay (PsVNA) using SARS-CoV-2 WA1/2020 strain and Omicron BA.1 subvariant. SARS-CoV-2 neutralizing activity measured by PsVNA correlates with PRNT (plaque reduction neutralization test with authentic SARS-CoV-2 virus) in previous studies^94, 95, 96^.

Neutralization assays were performed as previously described^13, 93, 95, 96, 97^. Brie y, 50 μL of SARS-CoV-2 S pseudovirions (counting ~200,000 relative light units) were pre-incubated with an equal volume of medium containing serial dilutions (starting at 1:10) of all samples at RT for 1 hr. Then, 50 μL of virus-antibody mixtures were added to 293T-ACE2-TMPRSS2 cells (10^4^ cells/50 μL) in a 96-well plate. The input virus with all SARS-CoV-2 strains was the same (2 × 10^5^ relative light units/50 μL/well). After a 3 hour incubation, fresh medium was added to the wells. Cells were lysed 24 hour later, and luciferase activity was measured using One-Glo luciferase assay system (Promega). The assay of each sample was performed in duplicate, and the 50% neutralization titer was calculated using Prism 9 (GraphPad Software). The limit of detection for the neutralization assay is 1:20. Two independent biological replicate experiments were performed for each sample and variation in PsVNA50 titers was <10% between replicates.

### High dimensional ow cytometry

#### SARS-CoV-2 specific B cell characterization with spectral ow cytometry

5 million cells per sample of PBMC, adenoid, or tonsil were resuspended in PBS with 2% FBS and 2 mM EDTA (FACS buffer). Biotinylated probes to SARS-CoV-2 were crosslinked with uorochrome-conjugated streptavidin in a molar ratio of 4:1. Fluorochrome-conjugated streptavidin was split into 5 aliquots and conjugated to biotinylated probes by mixing for 20 min/aliquot at 4 °C. Four uorochrome-conjugated probes were used in this assay, including spike S1 from the Wuhan strain (BioLegend) conjugated to APC, RBD from the Wuhan strain (BioLegend) conjugated to BV421, RBD from the Omicron variant (Acro) conjugated to BUV615, and RBD from the Omicron variant (Acro) conjugated to PE. Cells were first stained with the viability dye, Zombie NIR (1:800 dilution, BioLegend), for 15 min at RT, washed twice and then incubated with the 4 fluorochrome-conjugated probes plus d-biotin (Avidity) and Brilliant Stain Buffer Plus (BD Biosciences) at 4 °C for 1 hr. Then, cells were washed twice, and resuspended with True-Stain Monocyte Blocker (1:10 in 50 mL) (BioLegend) for 5 min. Anti-CXCR3 antibody and an antibody cocktail containing the rest of the surface antibodies (Supplementary Table 11) and Brilliant Stain Buffer Plus were sequentially added directly to the cells and incubated for 5 min and 30 min at RT, respectively (200 mL total staining volume). Cells were washed three times and xed in 1% paraformaldehyde for 20 min at RT before washing again and collecting on a spectral ow cytometer (Aurora, Cytek). Antibodies are listed in Supplementary Table 11. Gating strategies are shown in Supplementary Figure 1 – 2.

A separate panel including intracellular transcription factor staining was also used to stain PBMC and tonsil cells^92^. Antibodies are listed in Supplementary Table 11.

#### Immunophenotyping with 37 color spectral ow cytometry panel

Two million PBMCs per sample and 5 million cells per adenoid or tonsil were resuspended in FACS buffer after thawing. Cells were stained and acquired as described in our prior study^23^. Antibodies are listed in Supplementary Table 11. Manual gating for both panels was conducted with FlowJo Software v.10.9.0 (BD Biosciences) as in Supplementary Figure 3 – 4.

#### Unsupervised analysis of high dimensional ow cytometry data

S1^+^RBD^+^ B cells from the SARS-CoV-2-antigen-specific B cell panel with 29 parameters were analyzed with unsupervised clustering of surface antibody staining. S1^+^RBD^+^ live CD45^+^CD3^−^CD14^−^CD19^+^ B cells from INF and VAC groups were analyzed. Cells from tonsil and adenoid samples were merged and processed together, while PBMC samples were processed separately due to differences in cell populations. B cell analysis was based on surface expression of IgA, IgD, IgM, IgG, CD27, CD38, CD21, CD95, CD11c, FCRL3/FCRL5, FCRL4, CD10, CD86, CD83, CD69, CD71, CXCR3, CD85J, and CD62L. Channel values (scaled output with compensated parameters) of S1^+^RBD^+^ B cells from each sample were exported from FlowJo and then processed in R (v. 4.4.2) *via* Rstudio (2023.12.1+402). Each cell was given an index and labeled with its origin subject’s identification and tissue type. Data were further processed using Seurat^98, 99^(v. 5.1.0). Cell clustering was performed by applying the FindNeighbors() function^98^ on a distance matrix generated from the dist function by “euclidean” method, followed by Leiden clustering on the resulting SNN graph using Seurat’s FindClusters() algorithm, with a resolution parameter of 1.0 in PBMC and 0.6 in tissues. Expression of selected markers was visualized with their mean expression in each cluster by pheatmap (v. 1.0.12), and the downstream analysis and results were processed using ggplot2 (v. 3.5.1)^100^, reshape2 (v. 1.4.4)^101^, ggpubr (v. 0.6.0)^102^, ggthemes (v. 5.1.0)^103^ and tidyverse (v. 2.0.0).

### Statistical comparison with linear model

The majority of participants underwent tonsillectomy for obstructive sleep disordered breathing due to tonsillar hypertrophy (which includes those with obstructive sleep apnea diagnosed with polysomnography); a smaller portion had recurrent or chronic tonsillitis as their primary indication. We and others have noted that age and indication for tonsillectomy influence the immune cell populations in these tissues^56, 104^; however, none of the INF subjects had recurrent tonsillitis. This limitation prevented us from adjusting for this potential confounding factor. Sex had little effect in our cohort (evaluated with PCA analysis, data not shown).

Cluster proportion, cell percentages from manual gating, or neutralizing titers in each sample were modeled linearly as a function of (1) age (“Age”), and (2) history of SARS-CoV-2 infection or vaccine exposure (“Group” includes two or three categories: VAC, INF, and CON depending on the comparison. Each comparison involves two categories at a time.). The following formula was used to estimate separate coefficients for each category of Group (adjusted for age): lm (Frequency/Percentage ~Group + Age). To illustrate the extent of correction achieved by the linear model, both *p*-values from the two-sided Wilcoxon signed ranks test (in black) and p-values from linear model (in blue) were presented in plots built with ggplot2(v. 3.5.1)^100^.

### Single cell RNA sequencing

#### Sorting of S1^+^ and S1^−^ B cells for CITE-seq

We performed a combined analysis by merging data from three sets of cellular indexing of transcriptomes and epitopes (CITE-seq) experiments (B-2021, B-306–3 and B-306–4). B-2021 was performed and processed as reported in our prior study^23^. Brie y in B-2021, paired PBMC, adenoid, and tonsil samples from 3 donors (total of 9 donor-tissue samples) were assessed and ‘hashtag’ antibodies (BioLegend) were used to uniquely label each of the 9 donor-tissue samples^105^.

Two additional sets of CITE-seq experiments were conducted separately to include VAC samples (B-306–3 and B-306–4). For B-306–3, 2 INF and 1 CON tonsil were processed. For B-306–4, 3 VAC and 4 INF adenoid and 2 VAC, 2 INF, and 1 CON tonsil samples were processed as three sets (B-306–4 A, B, C) on 3 separate days over the course of one week to ensure manageable sorting times on each processing day (See Supplementary Table 8 for details on which samples were processed with each experiment). Frozen tonsil and adenoid cells were thawed from liquid nitrogen as described previously^23, 92^. For each donor, 100,000–500,000 thawed tonsillar cells were reserved for bulk RNA-seq. During data analysis, individual cells were demultiplexed using donor-specific single nucleotide polymorphism (SNP) information obtained from the bulk RNA-seq data (see Methods: CITE-seq processing and demultiplexing).

The remaining cells of the same tissue type from each donor were pooled together (i.e. adenoids and tonsils were pooled separately). The number of cells from each donor to pool was estimated using ow cytometry data with the aim of pooling a similar number of S1^+^ B cells from each sample. Pooled cells were incubated with Fc blocker at 4 °C for 10 min followed by CITE-seq and sorting antibody cocktails in the following order at 4 °C: TotalSeq anti-CXCR3, anti-CCR6, and anti-CXCR5 antibodies for 10 min, and the remaining 21 CITE-seq antibodies and uorescence-labeled sorting antibodies and viability dye (Aqua) for an additional 30 min (Antibodies are listed in Supplementary Table 11). Cells were then washed with PBS with 0.04% BSA and sorted on a BD FACS Aria^™^ III sorter (BD Biosciences, San Jose, CA). S1^+^ B cells from each tissue pool were sorted into a 200 uL low binding PCR tube (Eppendorf) with 50 mL culture medium (10% heat-inactivated FBS (VWR), 2 nM glutamine, 0.055 mM 2-mercaptoethanol, 1% penicillin/streptomycin, 1 mM sodium pyruvate, 10 mM HEPES, 1% non-essential amino acids in RPMI with glutamine), while S1^−^ B cells were sorted into 1.5 mL low binding tubes (Eppendorf) with 300 ml RPMI culture medium with 20% heat-inactivated FBS. All collecting low binding tubes were precoated with RPMI media with 20% FBS overnight at 4 °C prior to use. Sorting strategy is shown in Supplementary Figure 5A. Antibodies, including barcoded and fluorescence-conjugated antibodies, are listed in Supplementary Table 11. S1+ cells were centrifuged and resuspended for single cell partitioning without counting. An appropriate number of S1^−^ B cells were counted, aliquoted, centrifuged and resuspended for partitioning. Antibody concentrations used for CITE-seq were inferred based on the concentrations of antibodies of the same clones titrated for use in our ow cytometry staining of tonsil/adenoid cells.

### CITE-seq processing and demultiplexing

Library construction and sequencing for B-2021 were described previously^23^. For B-306–3 and B-306–4, sorted S1^+^ and S1^−^ B cells were mixed with the reverse transcription mix and partitioned into single cell gel-bead in emulsion (GEM) using 10× 5’ Chromium Next GEM Single Cell 5’ Reagent Kit v2 for B-306–3 and 1× 5’ Chromium Next GEM Single Cell 5’ HT v2 for B-306–4 (10× Genomics). The reverse transcription step was performed in an Applied Biosystems Veriti 96-well thermal cycler (Applied Biosystems). 5’ single cell gene expression (GEX), cell surface protein (CSP), and B cell receptor (BCR) libraries were prepared as instructed by 10x Genomics user guides (https://www.10×genomics.com/resources/user-guides/; CG000330 Rev F and CG000424 Rev C for the B-306–3 and B-306–4 run, respectively). Library quality and quantities were measured using a TapeStation system (Agilent) and a Qubit fluorometer (ThermoFisher). Libraries were pooled at a concentration of 10 nM and sequenced on the Illumina platform (NovaSeqX for B-306–3 and B-306–4, Illumina) using the following read lengths: Read 1: 26 base pairs, Index 1: 10 base pairs, Index 2: 10 base pairs, Read 2: 90 base pairs.

Tonsil cells saved for bulk RNA-seq from each participant were resuspended in QIAzol and RNA was extracted using RNeasy micro kit (Qiagen) and standard RNA sequencing libraries were generated using Universal Plus mRNAseq kit (TECAN Genomics). These libraries were used to generate SNP calls for each donor. Sequencing results were demultiplexed and converted to FASTQ format using Illumina bcl2fastq software. The sequencing reads were adaptor and quality trimmed and then aligned to the human genome using the splice-aware STAR aligner and SNP calls were called using bcftools^106^.

The pooled single cell RNA sequencing data was demultiplexed with a combination of demuxalot (v. 0.4.1)^107^ and demuxlet^108^ to match cells to each donor and identify doublets. For the pool with fraternal twin subjects, CNMC 124 and CNMC 125, cells were assigned by demuxalot, then reads with associated barcodes were extracted and reassigned via demuxlet to further re ne the cell identities for twin subjects.

### CITE-seq data analyses

Data from B-2021 set were processed as described^23^. For B-306–3 and B-306–4, CellRanger (v. 7.1.0)^109^ was used to map cDNA libraries to the GRCh38 genome reference (GRCh39–2020-A) and to count antibody tag features. Down-sampling was performed using the cellranger aggr pipeline (10× Genomics) to normalize sequencing depth across B cell lanes. Data were further processed using Seurat (v. 5.1.0) in R 4.4.2. Cells were demultiplexed by SNP as described above (Methods: Single cell CITE sequencing and demultiplexing). Surface protein library counts were transformed by using dsb (v. 1.0.4)^110^. For quality control, cells with less than 100 detected genes, greater than 30% mitochondrial reads, or gene counts greater than 25,000 were removed. To exclude cells with extremely high surface antibody counts, the top 0.5% of cells in the surface antibody total count distribution were removed. Cell clustering was performed by applying the FindNeighbors() function from Seuraton a distance matrix generated from the dsb-transformed surface protein data, followed by Leiden clustering on the resulting SNN graph using Seurat’s FindClusters() algorithm, with a resolution parameter of 0.3. Expression of selected genes were visualized using the ComplexHeatmap package (v. 2.22.0)^111^, and the percentage of cells per population for the S1^+^ and S1^−^ cells was plotted using ggplot2 (v. 3.5.2)^100^.

To further assess the transcriptome of B_SM_ subsets P1 to P4, we manually annotated B cell populations using protein expression data from CITE-seq for three reasons: (1) to align our analysis with ow cytometry data; (2) CXCR3 protein and mRNA expression levels were poorly correlated (Extended Data Figure 4a); and (3) to minimize batch effects across different experimental sets. We used the removeBatchEffect function from the limma (v. 3.62.1)^112^ package to correct batch effects in CSP expression within the B-306–4 set. CSP expression from B-2021, B-306–3, and B-306–4 were exported as .csv les and imported into FlowJo (v. 10.9.0) for manual gating. The gated FlowJo les were then processed using CytoML (v. 2.18.0)^113^ and flowWorkspace (v. 4.18.0)^114^, and the resulting gates were merged with the CITE-seq metadata by matching identical cell barcodes.

To identify differentially expressed genes among B_SM_ P1 to P4 (Extended Data Figure 4d), we used only the GEX from B-306–4 to minimize batch effects. Differential expression analysis was performed using the FindAllMarkers function in Seurat with the MAST algorithm, incorporating ‘Batch’ (defined as samples processed on different days within the B-306–4, set A-C), ‘Subject’, and ‘Tissue’ as latent variables (min.pct = 0.1, min.cells.feature = 3, min.cells.group = 3, logfc.threshold = 0.1). The PseudobulkExpression function was used to normalize count data for each B_SM_ population, yielding representative expression values for differentially expressed genes within each subset. The top 20 genes with adjusted p-values < 0.05 and log2FC > 0, as identified by the FindAllMarkers results, were visualized using pheatmap (v. 1.0.12)^115^.

#### Pseudo-bulk and differential gene expression analysis

Pseudo-bulk gene differential expression analysis and gene set enrichment analysis (GSEA) were performed as described previously^116, 117^. In brief, all sorted cells in a given cell type (S1^+^ or S1^−^) and tissue (adenoid or tonsil) within a donor were computationally ‘pooled’ according to their B cell subset assignment by summing all reads for a given gene. Pseudo-bulk libraries composed of only a few cells (less than 5), and with fewer than 30,000 unique molecular identifier counts after pooling per library were removed from the analysis, as they are likely not modeled properly by bulk differential expression methods. Only cell type- and tissue-specific B cell subsets with more than 3 psuedobulk libraries were included for differential comparison. Genes expressed at low levels were removed for each cell type individually using the lterByExpr function from edgeR^118^. Differentially expressed genes were identified using the limma voom^119^ work ow, which models the log of the counts per million (CPM) of each gene. Scaling factors for library size normalization were calculated with the calcNormFactors function with method = ‘RLE’.

Genes were ranked using the moderated T statistics for the relevant coefficient from the limma voom model. Differentially expressed genes between S1^+^ B_SM_ P1 and S1^+^ B_SM_ P2 cells were identified with a model accommodating paired analysis (formula: ~ 0+CD21_CXCR3+Subject_Tissue). The S1^+^ P1 and P2 B_SM_ subset (“CD21_CXCR3”) and tissue-specific subject (“Subject_Tissue”) were modeled as factor variable representing the B_SM_ population (including “B_SM_ P1” and “B_SM_ P2”), tissue type from a specific subject (including “tonsil” and “adenoid”), respectively. The contrasts. t function was then used to compare the estimated means between S1^+^ P1 B_SM_ and S1^+^ P2 B_SM_.

Enriched gene sets were identified using the pre-ranked gene-set enrichment analysis (GSEA) algorithm implemented in the fgsea (v. 1.32.0) R package. Gene set lists used for enrichment assessment (including Gene Ontology Biological Process (GO BP), GO Cellular Component (GO CC), GO Molecular Function (GO MF), Kyoto Encyclopedia of Genes and Genomes (KEGG), Reactome, the Molecular Signatures Database’s Hallmark collection, Blood Transcriptomic Modules and a few published datasets^59, 120, 121^) were collected and pooled. *P* values were adjusted using the Benjamini–Hochberg method for the whole gene set list. The pathways shown in Figure 5l were manually curated from gene sets relevant to immunology.

#### Signature scores of B cell subsets

Gene sets for atMBC were collected from studies on various chronic diseases in humans and mice including malaria^122^, HIV infection^123^, systemic lupus erythematosus^124^, Sjögren’s syndrome^125^, rheumatoid arthritis, common variable immunodeficiency^126^, and rodent malaria *Plasmodium chabaudi*^59^, as well as human tonsil^59^. Gene set for GCB signature was derived from a human tonsil B cell study^127^. cMBC signature was used from a recent study on human tonsil and murine malaria^59^. Other gene signatures used to characterize B cells were obtained from MSigDB^128^ (msigdbr R package, v. 7.5.1). The AddModuleScore function of Seurat was applied with default parameters to score each signature in each B cell. To represent the levels of each signature set in the populations of interest, the median score for each tissue- and subject-specific sample within the S1^−^ B cell subset from the B-306–4 set was used as an estimate. The median scores of per-subject median scores for each B cell population were then displayed in the heatmap of Figure 5j.

#### Trajectory analysis

To compare the lineage differentiation of S1^+^ B cells in INF and VAC tonsils and adenoids, we applied Slingshot (v. 2.14.0)^129^ to infer lineage trajectories. S1^+^ cells from B-306–4 set were used in this analysis. B cell subsets were assigned by manual gating as described in Figure 5 g and h. “PC” and “PreGC” cells were removed due to low number of cells for an accurate lineage estimation. First, uniform manifold approximation and projection (UMAP) embedding was performed on the normalized single-cell surface protein expression pro le from the CITE-seq dataset to obtain a low-dimensional representation. P1-P4 B_SM_ populations were provided as cluster input and “Naïve/USM B cell” population was appointed as the start cluster to Slingshot for trajectory reconstruction. Cells were ordered through inferred pseudotime based on gene expression to indicate their differentiation progress. Trajectories for INF and VAC groups were estimated separately.

#### BCR sequence analysis and clonal clustering

BCR repertoire sequence data were analyzed using the Immcantation (www.immcantation.org, v. 4.5.0) framework. Starting with ltered CellRanger output, V(D)J genes for each sequence were aligned to the IMGT GENE-DB reference database obtained 2/09/24 using IgBLAST (v. 1.22.0)^130^ and Change-O (v. 1.3.0)^131^. Nonproductive sequences, cells without associated constant region calls, cells identified as arising from doublets or negative droplets, and cells with multiple heavy chains were all removed. Samples within each subject were pooled and sequences were grouped into clonal clusters, which contain B cells related to each other by somatic hypermutation (SHM) from a common V(D)J ancestor. Using the hierarchicalClones function of scoper (v. 1.3.0)^132^, sequences within these groups differing by a participant-specific normalized Hamming distance threshold within the CDR3 region were defined as clones using single-linkage clustering (one subject required a slightly lower threshold than others)^133^. This threshold was determined by fitting a gamma/Gaussian mixture model to the distance to nearest sequence neighbor distribution using SHazaM(v. 1.2.0)^134^. These heavy chain-defined clonal clusters were further split if their constituent cells contained light chains that differed by V and J genes. Within each clone, germline sequences were reconstructed with D segment and N/P regions masked (replaced with “N” nucleotides) using the createGermlines function within dowser (v. 2.3)^135^. SHM was calculated as the frequency of non-ambiguous mismatches from each cell to the V gene (IMGT positions 1–312) of its reconstructed germline sequence. Paired scBCR-seq data were integrated with CITE-seq data based on matched cell barcodes.

To quantify B cell clonal diversity, we calculated Simpson’s diversity for each tissue-specific sample using the alphaDiversity function of alakazam (v. 1.3.0)^131^. Lower values of Simpson’s diversity indicate a greater probability of two random sequences belonging to the same clone, consistent with more large clones. To account for differences in sequence depth, samples within each comparison were down-sampled to the same number of sequences, and the mean of 1000 such re-sampling repetitions was reported. Subject/tissue/cell type samples or populations with < 70 B cells were excluded, which led to the exclusion of all S1^+^ cells from CNMC 10 and CNMC 99 (both CON with no history or evidence of SARS-CoV-2 infection or vaccination). Clonal overlap among tissues or B cell subsets can be used as a measure of immunological connectivity. Clonal overlap was calculated using the Jaccard index, which for each pair of B cell subsets is the number of unique clones found in both subsets (intersect) divided by the total number of unique clones among the two subsets (union). Clones were relabeled as “S1^−^” clone when the ratio of S1+ to S1− sorted B cells within the clone was less than 0.1. After clonal clustering, only heavy chain sequences were used for subsequent analysis. Clone expansion and B cell subset transition were estimated with indices (STARTRAC-expa and STARTRAC-tran) from the STARTRAC package (v. 0.1.0)^69, 70^. In STARTRAC-tran indices analysis, S1^+^ or S1^−^ subject specific populations with less than 10 cells were removed. Clones from INF and VAC groups were analyzed.

To infer lineage trees, we estimated tree topologies, branch lengths, and subject-wide substitution model parameters using maximum likelihood under the GY94 model^136, 137^. Using fixed tree topologies estimated from the GY94 model, we then estimated branch lengths and donor-wide parameter values under the HLP19 model in IgPhyML (v. 2.0.0)^136^. Trees were visualized using dowser (v. 2.3)^135^ and ggtree (v. 3.14.0)^138^.

### ATAC-seq

#### ATAC-seq data processing and analysis

Frozen tonsil cells were thawed and stained with antibodies listed in Supplementary Table 11 for 30 min at RT. Cells were washed with PBS twice and resuspended in RPMI with 10% FBS for sorting. 10,000 viable P1 (CXCR3^−^ CD21^+^), P2 (CXCR3^+^CD21^+^), P3 (CXCR3^+^CD21^−^) and P4 (CXCR3^−^CD21^−^) B_SM_ were sorted into 500 mL of FACS buffer using an FACSAria Fusion Sorter (BD) (cell sorting strategy is shown in Supplementary Figure 5B). Cells were pelleted and resuspended in 50 uL of transposase mixture including 25 mL 2×TD buffer (Illumina), 2.5 mL TDE1 (Illumina), 0.5 mL 1% digitonin (Promega) and 22 uL water. Tagmentation was performed by incubation at 37 °C for 30 minutes at 300 rpm. Following incubation, DNA was purified using a Qiagen MinElute kit, eluting samples in 10 uL. Purified tagmented DNA was PCR amplified using previously described primers^139^, with 12 cycles of amplification. Amplified libraries were purified using a Qiagen PCR cleanup kit and sequenced for 50 cycles (paired-end reads) on a NovaSeq 6000 (Illumina). ATAC-seq was done in three biological replicates per B_SM_ subset (samples shown in Supplementary Table 2). ATAC-seq primers are listed in Supplementary Table 11.

ATAC-seq data was processed using the chrom-seek pipeline (v. 1.0.0)^140^ with --assay ATAC (https://github.com/OpenOmics/chrom-seek). Reads were trimmed with Cutadapt (v. 4.4)^141^. All reads aligning to the Encode hg38 v1 blacklist regions^142^ were identified by alignment with BWA (v. 0.7.17)^143^ and removed with Picard SamToFastq. Remaining reads were aligned to an hg38 reference genome using BWA. Reads with a mapQ score less than 6 were removed with SAMtools (v. 1.17)^106^ and PCR duplicates were removed with Picard MarkDuplicates. Data was converted into bigwigs for viewing and normalized by reads per genomic content (RPGC) using deepTools^144^ (v. 3.5.1) using the following parameters: --binSize 25 --smoothLength 75 --effectiveGenomeSize 2805636331 --centerReads --normalizeUsing RPGC. Averaged bigwigs were created using the bigwigAverage function of deepTools (v. 3.5.4)^144^.

Peaks were called using macsNarrow^145^ (macs v. 2.2.7.1) with the following parameters: -q 0.01 –keep-dup=“all” -f “BAMPE”. Differential peaks were called using DiffBind (v. 2.15.2)^146^ and its Deseq2 differential caller with default parameters. Peaks were considered significant with an FDR value less than 0.1. Motif analysis was completed using the MEME suite (v. 5.5.5)^147^. Known motif enrichment analysis was accomplished using AME on a combined jolma 2013, jaspar 2018 core vertebrate non-rendundant, and HOCOMOCO (v.11)^148^ full human mono database. Downstream analyses and results visualization were performed with R (v.4.4.2) and visualized with ggplot2 (v. 3.5.1)^100^.

#### Differentially accessible regions (DAR) and pathway enrichment analysis

Differential peaks from comparing P1 B_SM_ and P2 B_SM_ (adjusted p-value < 0.10) were selected for downstream analysis. To explore the functional significance of these DARs, pathway enrichment analysis was conducted using GREAT (Genomic Regions Enrichment of Annotations Tool, v. 4.0.4)^149, 150^. The genomic coordinates of DARs were converted to BED les and uploaded to the GREAT web server. The analysis was performed mapping to GRCh38 (UCSC hg38, Dec. 2013) and using the default association rules, which map genomic regions to nearby genes based on a basal plus extension model (5 kb upstream, 1 kb downstream, and up to 1 Mb extension from the transcription start site). Enriched terms from GO BP and GO MF, MSigDB, Reactome, and other curated databases were extracted. Pathways and terms with an FDR (adjusted *p*-value) < 0.05 were considered significant. Top20 GO BP pathways ranked by FDR (adjusted *p*-value) values were visualized with bar plot.

#### IRF4 complex motif search with FIMO

To further evaluate the enrichment of IRF4 complex motifs in P1 B_SM_ and P2 B_SM_, a search with stringent EICE (GGAANNGAAA), ISRE (A/GNGAAANNGAAACT) and two AICE (0bp/4bp: 0bp – GAAATGA(G/C)TCA; 4bp – TTTCNNNNTGA(G/C)TCA)^151^ motifs using Find Individual Motif Occurrences (FIMO) on the DAR sequences obtained from P1 B_SM_ to P2 B_SM_ comparison was performed^67^. The enrichment statistics were calculated as above using a two-tailed version of Fisher’s exact test.

For Extended Data Figure 4i, data for memory B cells (MBC) and plasma cells (PC) were retrieved from a published dataset (https://zenodo.org/records/8373756)^99^ and loaded into Signac (v1.11.0)^152^. Plots were created with the Signac CoveragePlot function.

### BCR signaling assessment by phosphorylation staining

Tonsil cells were thawed, rinsed with RPMI supplemented with 0.1% FBS, and rested for 80 minutes at 37 °C in the same medium. After resting, the cells were incubated with a live/dead stain in PBS for 15 minutes at RT and then washed once with PBS. This was followed by staining the cells with antibodies against CXCR3 and CXCR5 for 5 minutes at RT. Subsequently, the remaining surface antibody mix was added (Supplementary Table 11), and the cells were resuspended in FACS buffer for 20 minutes at RT^92^. Afterward, the cells were washed twice with RPMI supplemented with 0.1% heat inactivated FBS and then resuspended in pre-warmed RPMI with 10% FBS and stimulated with anti-BCR antibodies, as previously described^71, 153, 154^. Stimulation was carried out at 37 °C for 2 minutes using 10 mg/mL goat F(ab’)2 anti-human IgA/G/M (Jackson ImmunoResearch Laboratories). For detecting phosphorylated signaling intermediates, the cells were fixed and permeabilized using BD Cytofix and Phosflow Perm/Wash buffers (BD Biosciences), then stained with PE-phosphorylated Syk (p-Y348) and Alexa Fluor 488- phosphorylated PLCg2 (p-Y759) antibodies (BD Biosciences). Samples were acquired on an Aurora cytometer (Cytek), and analysis was performed using FlowJo (v. 10.9.0). Gating strategies are shown in Supplementary Figure 6A.

### *In vitro* plasmablast differentiation and proliferation assay

Tonsil cells were thawed and stained with viability dye and surface antibody mix (Supplementary Table 11) at RT for 30 mins. Then cells were washed with PBS twice and resuspended in RPMI with 10% FBS at concentration of 5 million cells/mL for sorting. P1, P2, P3 and P4 B_SM_ were sorted on Aria sorter (BD) into 0.3 mL RPMI with 20% FBS in 1.5 mL tubes (Supplementary Figure 5B). Sorted cells were centrifuged at RT for 10 mins and then were labeled with 0.5 mM CFSE (CellTrace CFSE cell proliferation kit, ThermoFisher) in 1100 μL PBS at RT in the dark for 10 mins. Then, cells were washed with pre-warmed RPMI + 10% FBS as described^153^. Allogenic B cell-depleted PBMCs were prepared using a B cell depletion kit (Dynabeads CD19 Pan B, ThermoFisher) from PBMCs of an unrelated healthy donor. The sorted memory B cells and allogeneic B cell depleted PBMC were co-cultured at a 1:9 to 2:8 ratio with 2.5 mg/mL R848 and 1000 U/mL recombinant human IL-2 for 4 days at 1 × 10^6^ cells per well in a 96-well at bottom plate. RPMI with 10% FBS was used for culture. The cells were collected and stained with antibodies against CD19, CD20, CD3, CD27, CD21, IgD, CD38, and CXCR3, fixed (Lysing Solution, BD Biosciences), permeabilized (Permeabilizing Solution 2; BD Biosciences) and stained with antibodies against IgG, IgA, IgM. The cells were acquired on an Aurora cytometer (Cytek) and the analyzed using FlowJo (v. 10.9.0). Antibodies are listed in Supplementary Table 11. Gating strategies are shown in Supplementary Figure 6B. The division index, a measure of the overall proliferative response, is the average number of divisions undergone per cell in the total population, including cells that have not undergone division.

### Tissue processing and staining for immunofluorescence assay

Formalin-fixed, paraffin-embedded (FFPE) adenoid and tonsil tissue blocks were cut into 5 μm sections and mounted onto charged slides. Two paired tonsil and adenoid samples (from one INF donor and one VAC donor) were loaded onto the same slide. For staining, slides were deparaffinized and tissue rehydrated in deionized water. Antigen retrieval was performed by incubating slides in antigen retrieval (AR) buffer (Cepham life sciences) for 45 min in a steamer (preheated, approximately 95 °C). After 45 min, slides were taken out from the steamer and allowed to cool to RT. Sections were permeabilized, blocked for 1 hour in PBS containing 0.3 % Triton X-100 (Sigma-Aldrich), 1% bovine serum albumin (Jackson Immune Research). Sections were stained with titrated amounts of non-conjugated primary antibodies, followed by overnight incubation at 4 °C. Slides were then washed with PBS (3 times, 10 min each) and stained with the appropriate secondary antibodies for 2 hrs at RT. Slides were washed and blocked again for 1 hr at RT with a 1:10 dilution of normal mouse and rabbit or goat serum. Then, slides were stained with titrated amounts of directly conjugated antibodies for 2 hrs at RT. After three final washing steps and staining with the nuclear marker TOPRO (ThermoFisher), slides were mounted with prolonged gold anti-fade mounting media (ThermoFisher) and sealed with a glass coverslip. Antibodies are listed in Supplementary Table 11.

Tissue sections were imaged (using confocal system, Leica Stellaris RTB WLL FLIM) as three-dimensional (3D) tile scans and subsequently mosaic-merged to generate a continuous representation. To minimize imaging artifacts, corrections were applied for motion-induced distortions, 3D alignment inconsistencies, and thermal drift across sequential z-sections. Additionally, crosstalk and color calibration adjustments were performed using Huygens Pro (version 24.04.0p3 64-bit, Scientific Volume Imaging BV). Image deconvolution was conducted within the same software to enhance signal resolution.

The reconstructed images were further processed using Imaris (v. 10.2.0, Oxford Instruments). A combination of colocalization analysis, ChannelArithmetics, Xtension, Imaris installed machine learning-based classification, and masking techniques were employed to delineate distinct anatomical regions as additional computational channels, including follicles, germinal centers, extrafollicular regions, epithelium, and crypt structures. The Surface module of Imaris was utilized to generate cell objects, integrating nuclear signals and perinuclear regions derived from the preceding image processing steps.

Quantitative data were extracted from processed les *via* a custom parser script, which reformatted surface object statistics into “.csv” les optimized for direct import into FlowJo (v. 10.9.0) for gating and further analysis. Statistical analysis and visualization were further processed in GraphPad Prism (v. 10.2.0392).

### SPACE analysis of immunofluorescence imaging data

Spatial Patterning Analysis of Cellular Ensembles (SPACE) is an R package designed to identify complex spatial patterns at the cell and tissue levels^79^. Cell objects from immunofluorescence images were annotated with 8 populations as in Figure 8f by manual gating with FlowJo (v. 10.9.0) and labeled by an R-based customized script. The 10 μm radius captures close cellular associations and was chosen for SPACE “census_image” function. The number of neighborhoods was chosen to achieve 5 × tissue coverage. Covariation plots were created using the SPACE “learn_pattern” function.

### Spatial transcriptomic pro ling with Xenium In Situ platform

Slides were prepared following the manufacturer’s instructions and work ow for FFPE tissue samples (CG000578 Rev A; 10× Genomics). A 5-μm section from the tissue block containing the same paired tonsil and adenoid samples (one from INF donor and one from VAC donor) used for immunofluorescence were carefully attached to the sample area on a Xenium slide (Histoserv, MD).

Samples are listed in Supplementary Table 2. Xenium slides were deparaffined and rehydrated and were then assembled into the Xenium Cassette. Deparaffinized slides in the Xenium cassette were decrosslinked (CG000580 Rev A) and immediately underwent probe hybridization, ligation, and amplification (CG000582 Rev D) using the Xenium 5000 human gene panel (Prime 5K Human Pan Tissue & Pathways Panel). With auto fluorescence quenching and nuclei staining, the tissue images were captured and analyzed by the Xenium Analyzer (PN-1000569, instrument software v2.0.1.0). Regions of interest were manually selected from the scanned images. Post-run data for each slide was obtained using default parameters for downstream analysis.

Data were processed by Xenium analysis software (v 2.0.0.10). The raw count matrix was pre-processed using the Seurat package (v. 5.1.0)^98, 99^ in R (v. 4.4.2). For quality control, cells with at least nCount > 40, 15 nFeature > 10, cell_area > 10 & < 200 were retained^155^. Raw count data were normalized using the SCTransform function with method “glmGamPo ”. Dimension reduction was performed using the runPCA function and the optimal number of principal components was selected using the ElbowPlot function. Cell clusters were determined using the FindClusters function.

We annotated cell populations with human tonsil reference version 2 with Seurat (v. 5.0.2)^98^ and Azimuth (v. 0.5.0)^91, 99^. Cell populations with less than 150 cells per slide were removed (granulocytes, mast, preB/T and PC/doublet cells). Some cell types were merged (B naïve includes B naive & B activated; B memory includes B memory & FCRL4/5^+^ B memory; CD4 Non-TFH includes CD4 Non-TFH & CD4 TCM; CD4 TFH includes CD4 TFH & CD4 TFH Mem; CD8 non-naïve includes CD8 T & CD8 TCM; gdT_MAIT includes MAIT/TRDV2 + gdT & non-TRDV2 + gdT; Mono/Macro includes Mono/Macro & Cycling myeloid; PB/PC includes PB & PC).

### Additional statistical analyses

Correlations were analyzed using Spearman’s rank correlation test using base R and corrplot (v 0.95). Paired comparisons were performed with Wilcoxon signed ranks test with ggpubr (v. 0.6.0)^156^ and visualized with ggplot2 (v. 3.5.1)^100^ in R (v. 4.4.2).

## Supplementary Material

Supplementary Files

This is a list of supplementary les associated with this preprint. Click to download.

- 20250805SupplementaryTable110.xlsx
- 20250805SupplementalTable11.xlsx
- 20250813SupplementaryFigures.docx
- EXTENDEDDATAFIGURES.docx

## Data Availability

All data necessary to understand and evaluate the conclusions of this paper are provided in the article and Supplementary Information. The CITE-seq and ATAC-seq data will be deposited to dbGAP (the study is in the process of getting registered). Source data will be provided with the final published manuscript.

## Code availability:

The R scripts used in this paper are available on a GitHub repository at https://github.com/kalpanamanthiram/COVID-19-Vaccination.
